# Metabolic reprogramming of hepatocytes by *Schistosoma mansoni* eggs

**DOI:** 10.1016/j.jhepr.2022.100625

**Published:** 2022-11-07

**Authors:** Verena von Bülow, Sarah Gindner, Anne Baier, Laura Hehr, Nicola Buss, Lena Russ, Sarah Wrobel, Victoria Wirth, Kuscha Tabatabai, Thomas Quack, Simone Haeberlein, Patrik Kadesch, Stefanie Gerbig, Katja R. Wiedemann, Bernhard Spengler, Annabel Mehl, Gertrud Morlock, Gabriele Schramm, Jörn Pons-Kühnemann, Franco H. Falcone, R. Alan Wilson, Katrin Bankov, Peter Wild, Christoph G. Grevelding, Elke Roeb, Martin Roderfeld

**Affiliations:** 1 Department of Gastroenterology, Justus Liebig University, Klinikstr. 33, 35392 Giessen, Germany; 2 Institute of Parasitology, BFS, Justus Liebig University, Schubertstr. 81, 35392 Giessen, Germany; 3 Institute of Inorganic and Analytical Chemistry, Justus Liebig University, Heinrich-Buff-Ring 17, 35392 Giessen, Germany; 4 Institute of Nutritional Science, Food Science Department, and Interdisciplinary Research Center (iFZ), Justus Liebig University Giessen, Heinrich-Buff-Ring 26-32, 35392 Giessen, Germany; 5 Experimental Pneumology, Priority Research Area Asthma & Allergy, Research Center Borstel, Parkallee 1-40, 23845 Borstel, Germany; 6 Institute of Medical Informatics, Justus Liebig University, Rudolf-Buchheim-Str. 6. 35392 Giessen, Germany; 7 York Biomedical Research Institute, Department of Biology, University of York, York YO10 5DD, UK; 8 Dr. Senckenberg Institute of Pathology, University Hospital Frankfurt, Frankfurt am Main, Germany

**Keywords:** Schistosomiasis, Parasite, Oxidative stress, Lipid, DNA damage, bs, bisex, DMPE, dimethyl-phosphatidylethanolamine, flOA, fluorescently labelled OA, GSH, reduced L-glutathione, GS, glycogen synthase, HCC, hepatocellular carcinoma, *hR*_F_, retention factor ∗ 100, MALDI-MSI, matrix assisted laser desorption/ionization mass spectrometry imaging, MDA, malondialdehyde, ms, monosex, ni, non-infected, OA, oleic acid, PAS, periodic acid-Schiff, PC, phosphatidylcholine, PDH, pyruvate dehydrogenase, PE, phosphatidylethanolamine, PLIN2, perilipin 2, ROS, reactive oxygen species, SEA, soluble egg antigens, *S. japonicum*, *Schistosoma japonicum*, *S. mansoni*, *Schistosoma mansoni*, TG, triglyceride

## Abstract

**Background & Aims:**

Schistosomiasis is a parasitic infection which affects more than 200 million people globally. *Schistosome* eggs, but not the adult worms, are mainly responsible for schistosomiasis-specific morbidity in the liver. It is unclear if *S. mansoni* eggs consume host metabolites, and how this compromises the host parenchyma.

**Methods:**

Metabolic reprogramming was analyzed by matrix-assisted laser desorption/ionization mass spectrometry imaging, liquid chromatography with high-resolution mass spectrometry, metabolite quantification, confocal laser scanning microscopy, live cell imaging, quantitative real-time PCR, western blotting, assessment of DNA damage, and immunohistology in hamster models and functional experiments in human cell lines. Major results were validated in human biopsies.

**Results:**

The infection with *S. mansoni* provokes hepatic exhaustion of neutral lipids and glycogen. Furthermore, the distribution of distinct lipid species and the regulation of rate-limiting metabolic enzymes is disrupted in the liver of *S. mansoni* infected animals. Notably, eggs mobilize, incorporate, and store host lipids, while the associated metabolic reprogramming causes oxidative stress-induced DNA damage in hepatocytes. Administration of reactive oxygen species scavengers ameliorates these deleterious effects.

**Conclusions:**

Our findings indicate that *S. mansoni* eggs completely reprogram lipid and carbohydrate metabolism via soluble factors, which results in oxidative stress-induced cell damage in the host parenchyma.

**Impact and implications:**

The authors demonstrate that soluble egg products of the parasite *S. mansoni* induce hepatocellular reprogramming, causing metabolic exhaustion and a strong redox imbalance. Notably, eggs mobilize, incorporate, and store host lipids, while the metabolic reprogramming causes oxidative stress-induced DNA damage in hepatocytes, independent of the host's immune response. *S. mansoni* eggs take advantage of the host environment through metabolic reprogramming of hepatocytes and enterocytes. By inducing DNA damage, this neglected tropical disease might promote hepatocellular damage and thus influence international health efforts.

## Highlights

• *S. mansoni* soluble egg products induce hepatocellular metabolic reprogramming.
• *S. mansoni* eggs mobilize, incorporate, and store host lipids.
• *S. mansoni* provoke parenchymal reprogramming and cause metabolic exhaustion in the liver.
• *S. mansoni* eggs prompt a metabolic crisis-associated redox imbalance and provoke DNA damage.
• *S. mansoni* eggs induce processes that harm hepatocytes independent of the host's immune response.

## Graphical abstract

**Figure:**
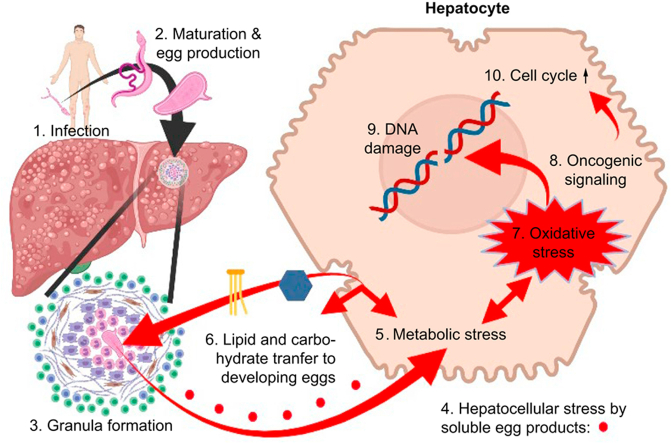

## Introduction

Schistosomiasis, one of the most important parasitic infections worldwide, is caused by trematodes of the genus Schistosoma. It is accompanied by severe clinical symptoms as well as socioeconomic problems, and more than 200,000 deaths per year. According to the World Health Organization (WHO), schistosomiasis is a neglected tropical disease, and at least 236.6 million people required preventive treatment in 2019.^1^ Climate change and globalization will inevitably influence both the distribution of parasites and the incidence of schistosomiasis, even in areas where it is currently not endemic. Reports about an outbreak of urogenital schistosomiasis in Corsica (France) demonstrate the potential risk of schistosomiasis spreading into non-endemic areas.^2^

In intestinal schistosomiasis, schistosome eggs cause inflammatory responses leading to granuloma formation.^3^ The process of egg excretion is driven by an immune-dependent formation of granulomatous inflammation in the gut. A similar immunopathology is caused in the hepatic dead-end.^4^ In contrast to intestinal granulomas, liver granulomas become fibrotic over time, which often leads to obstructive portal lesions and portal hypertension, resulting in gastrointestinal bleeding, hepatic encephalopathy, or liver failure.^4^

There is compelling evidence that schistosome eggs, but not the adult worms, are mainly responsible for schistosomiasis-specific morbidity.^5^ Unusually for trematodes, *S. mansoni* eggs are not self-contained. As a central part of the eggs, the supporting vitelline cells are unable to drive embryonic development, which also depends on host factors. Taking into account density and volume changes between deposition and maturation, it has been estimated that an egg increases in mass by more than threefold.^6^ This means that more than two-thirds of tissue constituents in a mature egg are externally derived, while even neglecting energy requirements during embryogenesis.^6^

In endemic areas, *S. mansoni* infection is associated with anemia and chronic malnutrition.^7^ It is unclear if schistosomiasis is a cause of anemia and malnutrition or if both are threatening consequences of the poverty-related living conditions in endemic areas. Nevertheless, malnutrition is one of the major factors driving schistosomiasis.^8^ The current study illuminates the physiologic consequences of an *S. mansoni* infection, thus improving our understanding of the connections between malnutrition and schistosomiasis.

In contrast to *S. mansoni*-associated malnutrition in endemic areas, positive metabolic effects of infection have been shown in humans suffering from the metabolic syndrome.^9^ Furthermore, the injection of *S. mansoni* eggs into mice indicated that the cholesterol-lowering effect observed in the serum during infection is mediated by factors released from eggs, while adult worms seemed to have little or no effect.^10^ *S. japonicum* infection induced host genes involved in catabolism including glucose uptake, glycolysis, fatty acid oxidation, and suppression of anabolism (glycogen synthesis) in the liver. This might be regulated by macrophage metabolic states involving AMPK (AMP-activated protein kinase), AKT, and TORC1 (target of rapamycin kinase complex I) pathways, as shown by *in vitro* stimulation with soluble egg antigens (SEA: representing the total soluble complement of homogenized eggs).^11^ A recently published study demonstrated reprogramming of the metabolic signature of macrophages after *S. mansoni* infection.^12^ Of note, this reprogramming paralleled the establishment of a longevity memory-like phenotype in myeloid cells of infected mice.^12^

Imbalances of nutrition supply, *i.e.* undernutrition as well as overnutrition, have been associated with enhanced hepatic oxidative stress that promotes liver disease.^13^ Increased oxidative stress during *S. mansoni* infection has been attributed to the inflammatory response of granulomatous immune cells within the liver.^14^ High reactive oxygen species (ROS) levels induce oncogenic signaling and may promote cancer by increasing DNA mutations.^15^ Previously, we discovered that SEA, including IPSE/alpha-1 of *S. mansoni*, activates oncogenic signaling in the liver and colon.^16^^,^^17^

In this study we provide the first evidence that *S. mansoni* eggs are capable of inducing metabolic reprogramming of the host parenchyma, in order to take up host metabolites. These processes involve hepatic exhaustion, finally leading to hepatic DNA damage.

## Materials and methods

### Human material

Pseudonymized human colon samples were kindly provided by Dr. Senckenberg Institute of Pathology, University Hospital Frankfurt. The use of pseudonymized human residual samples that were routinely taken for pathologic assessment was approved by the local ethics committee (AZ 05/19). According to the ethics vote, informed consent was not required for our retrospective analyses of archived tissues.

### Animal experimentation

*Biomphalaria glabrata* snails served as intermediate hosts and Syrian hamsters (*Mesocricetus auratus*) as final hosts for maintaining the life-cycle of a Liberian strain of *S. mansoni*.^18^ Bs (= mixed sex) and ms (= monosex) worm populations were generated by polymiracidial and monomiracidial intermediate host infections, respectively.^19^ Bs infections (n = 36 ♀) were carried out at the age of 8 weeks and were maintained for 46 days, and ms infections (n = 17 ♀) 67 days^20^ to ensure a complete maturation of the worms; females need longer to grow and develop in the absence of male partners. Untreated hamsters (n = 6 ♀) were used as supercontrols. All animal experiments were performed in accordance with the European Convention for the Protection of Vertebrate Animals used for experimental and other scientific purposes (ETS No 123; revised Appendix A) and were approved by the Regional Council Giessen (V54-19 c 20/15 c GI 18/10 Nr. A26/2018).

### MALDI-MSI analysis and identification by liquid chromatography-tandem mass spectrometry

Matrix-assisted laser desorption/ionization mass spectrometry imaging (MALDI-MSI) and liquid chromatography-tandem mass spectrometry experiments were performed as described elsewhere.^21^ For data analysis, ”Lipid Match Flow”^22^ was used for identification, ”Perseus”^23^ for statistical analysis and ”Mirion”^24^ for image generation. Further details are provided in the supplementary information.

### Isolation of soluble egg antigens

*S. mansoni* eggs were obtained from livers of bs-infected hamsters at day 46 post infection, and SEA were isolated as described previously.^25^

### Isolation of *S. mansoni* eggs

Liver eggs and *in vitro*-laid eggs of *S. mansoni*-infected hamsters were isolated as described elsewhere with minor modifications.^25^^,^^26^

### Cell culture experiments

HepG2 cells (stock ordered in 2019, CLS # 330198, expanded and stored as cryostocks for consistent quality in culture for up to 10 passages per cryostock) were stimulated with 15 μg/ml SEA and/or 10 mM reduced L-gluthatione (GSH) for the indicated time points. For co-culture assays, HepG2 cells were treated with 400 μM fluorescently labeled oleic acid (Avanti Polar Lipids, Alabaster, AL, USA, #810259C) for 24 h and washed three times with PBS prior to co-culturing with 100 *S. mansoni* eggs per well (24-well plate) for a further 24 h. *In vitro*-laid or liver-extracted eggs were co-cultured in the same compartment or in transwells with a permeable polycarbonate membrane (Corning, New York, USA), as indicated in the figure legends. Subsequently, eggs were washed three times and analyzed directly or after fixation with 1% formaldehyde by confocal laser scanning microscopy.

### Confocal laser scanning microscopy and live cell imaging

For lipid staining of eggs, fluorescently labeled oleic acid (flOA; TopFlour Oleic Acid, Avanti SKU 810259C) was applied and uptake analyzed by confocal laser scanning microscopy. Liver eggs or *in vitro*-laid eggs were cultured up to 24 h with 50 μg/ml flOA (mixed with 200 μM oleic acid) or with HepG2 cells pretreated with 50 μg/ml flOA (mixed with 200 μM oleic acid) 24 h prior to co-culture with eggs. Negative controls received oleic acid only. Image acquisition was done on a Leica TCS SP5 VIS using a 488 nm argon laser. Autofluorescence of eggs was excited with a DPSS laser at 561 nm to visualize egg contours. Live imaging of flOA uptake was conducted by image acquisition every 20 s over a period of 30 min. Confocal z-stacks were composed of 100-122 images with a step size of 0.5 μm and further processed in IMARIS imaging software (Bitplane). Quantification of flOA fluorescence per egg was achieved by acquiring 15-25 z-stacks and averaging the mean grey values of the egg in all images using Image J.

### Histochemistry and immunohistochemistry

Periodic acid-Schiff (PAS) reaction was used to visualize glycogen distribution in hepatic tissue. To analyze the hepatic distribution of neutral triglycerides (TGs) and lipids, liver tissue was stained with the fat-soluble Oil red O dye (Serva, Heidelberg, Germany, #31170). Immunohistochemical detections were performed as described previously.^27^

### TG assay

The TG concentration of homogenized hamster liver lysates was determined as recommended by the manufacturer (Triacylglyceride Assay Kit – Quantification, Abcam, Cambridge, UK, #ab65336).

### High-performance thin-layer chromatography

The prepared liver tissue extracts were ten times more concentrated than previously described.^28^ Instrumentation used was from CAMAG, Muttenz, Switzerland. Samples and calibration standards (1 mg/ml in chloroform/methanol 3:1, v/v) were applied as 8 mm bands (ATS4) on high-performance thin-layer chromatography (HPTLC) silica gel 60 F_254_ MS grade plates (Merck, Darmstadt, Germany) and dried (40 °C, 5 min). Separation of polar lipids was performed with chloroform/methanol/ammonia (25%)/water 60:30:3:1 (v/v/v/v) up to 50 mm, and of non-polar lipids with *n*-hexane/diethyl ether/acetic acid 40:10:1 (v/v/v) up to 65 mm migration distance after 20 min pre-saturation (via filter paper, Twin-Trough Chamber). The dried plate (5 min, cold air stream) was immersed into the primuline reagent (0.05 % in acetone/water 4:1, v/v). Fluorescence detection was at 366/>400 nm (TLC Scanner 4). For visualization of saccharides and amino acids per reagent sequence, the primuline-treated polar lipid plate was immersed into the ninhydrin and then aniline-diphenylamine-*o*-phosphoric acid reagents.^29^ The primuline-treated non-polar lipid plate was sprayed with phosphomolybdic acid reagent (20 mg/ml solution in ethanol), heated (110 °C, 3 min) and detected at white light illumination. For high-resolution mass spectrometry (HRMS), samples and standards were applied in duplicate on prewashed plates,^30^ which were cut after development. One plate part was used for detection via the primuline reagent, and the other via HPTLC–HRMS as described.^28^

### Reporter gene assay

Analysis of AP-1 promoter activity in SW620 cells has been described previously.^16^

### Western blot analysis

Western blotting was performed as described previously.^31^

### Glycogen assay

Liver tissue and glycogen standard samples were dissolved in 2 M hydrochloric acid, boiled for 1 h and neutralized with 2 M sodium hydroxide. After centrifugation, the supernatant was analyzed with the Glucose Assay Kit (Merck) according to the manufacturer's protocol.

### Malondialdehyde assay

Lipid-peroxidation as a marker for oxidative stress was measured in liver samples and HepG2 cells by the malondialdehyde (MDA) Assay Kit (Merck), according to the manufacturer's protocol.

### Pyruvate assay

The pyruvate content of HepG2 cells was determined by the Pyruvate Assay Kit (Merck) according to the manufacturer's protocol.

### Quantitative real-time PCR

mRNA isolation, transcription, quantitative real-time PCR, and data analysis were performed as described previously.^32^

### Quantification of *S. mansoni* eggs

Liver tissue (100 mg) of bs-infected hamsters (n = 36) was digested in 5% potassium hydroxide at 37 °C for 16 h.^33^ Subsequently, eggs were counted independently by two persons three times per sample. The number of eggs was calculated per mg of liver tissue.

### Comet assay

The alkaline comet assay (Abcam #ab238544) for assessing DNA damage in HepG2 cells was performed according to the manufacturer's protocol.

### Statistical analysis

The present study is of an exploratory nature. Therefore, the group sizes were not estimated in advance by pre-specified effect sizes. The study was started with an existing number of cryopreserved organs that were not required for the maintenance of the parasite life cycle. Statistical analysis was performed using SPSS version 26.0 (SPSS Inc., IBM corporation, Armonk, NY, RRID:SCR_002865). Subsequent comparison of groups within each Kruskal-Wallis test were Bonferroni-corrected. Because of the exploratory nature of the study, no further adjustment for *p* values was performed. Densitometrically assessed data from western blots (hamster colon) were depicted as mean or median ± 95% CIs.

## Results

### *S. mansoni* eggs disrupt hepatic lipid metabolism

**Fig. 1:**
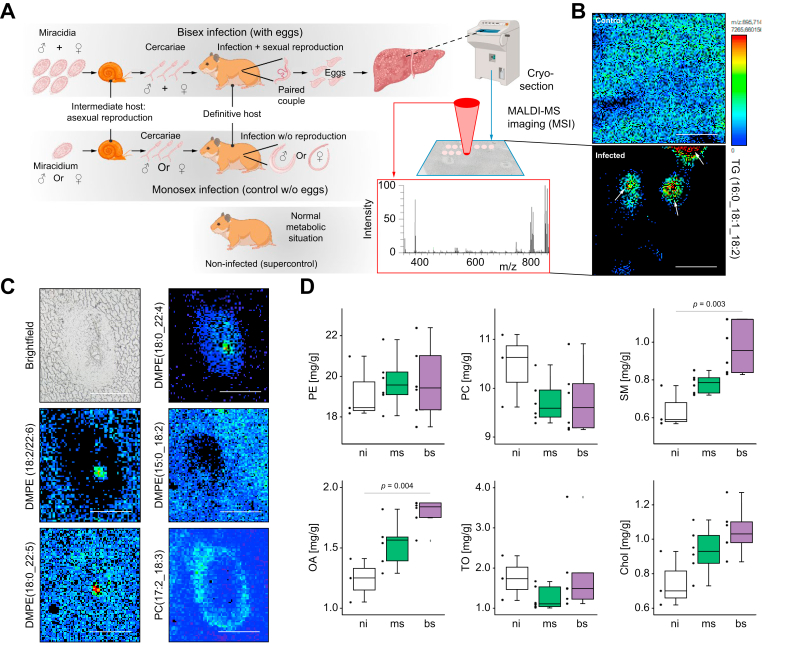
Infection with *S. mansoni* induces alterations in the composition and distribution of hepatic lipids. (A) Infection of hamsters with *S. mansoni* cercariae of both sexes (bs infection) or with clonal cercariae of one sex (ms infection) in order to compare egg-induced *vs.* worm-only effects. The distribution of lipids was analyzed by MALDI-MSI in three biological replicates of bs-infected- and ms-infected hamster, and ni (non-infected) control cryosections, respectively. (B) *S. mansoni* infection causes an accumulation of TGs in the granuloma area with highest levels inside the eggs (arrows). TGs were depleted in the surrounding liver tissue compared to ni controls. The panels depict the distribution of TG(16:0_18:1_18:2)+K, m/z 895.714876 in the liver of bs-infected-hamster and ni controls. (C) The distribution of lipid species differs characteristically in eggs, granuloma, and the areas surrounding granulomas. Upper left: Brightfield image. Middle left: DMPE (18:2/22:6) was found depleted in granulomas and enriched in eggs. Lower left: DMPE (18:0/22:5) was also detected with higher intensity in the eggs, but no depletion was observed for the granuloma regions. Upper right: DMPE (18:0/22:4) was mainly found in granulomas with slight enrichments in eggs. Middle right: DMPE (15:0/18:2) was found depleted in some granulomatous regions. Lower right: PC(17:2_18:3) [M+HCO_2_]^-^, m/z 810.532050, was found enriched in the outer borders of granulomas. A second set of MSI-pictures demonstrating altered lipid distribution in *S. mansoni*-infected hamsters is depicted in Fig. S5. These experiments were performed at least three times independently. (D) The quantification of selected lipid species revealed enhanced hepatic levels of SM and OA in bs-infected animals. These experiments were performed at least three times independently. Levels of significance are indicated in the figure (Kruskal-Wallis test). bs, bisex; Chol, cholesterol; DMPE, dimethylphosphatidylethanolamine; MALDI-MSI, matrix assisted laser desorption/ionization mass spectrometry imaging; ms, monosex; ni, non-infected; OA, oleic acid; PC, phosphatidylcholine; SM, sphingomyelin; TG, triglyceride; TO triolein.

To study the role of *S. mansoni* eggs in the liver *in vivo*, we used a hamster model and infections with bisex (bs)- and monosex (ms)-cercariae. This allowed us to discriminate between worm-induced and egg-mediated effects *in vivo* (Fig. 1A). For analyzing the hepatic distribution of certain lipid species in livers of bs-infected hamsters, we performed MALDI-MSI,^34^ revealing the relative abundance and locations of hepatic lipids (Fig. 1A). MSI experiments revealed that all detected TGs accumulated in the granuloma area, with the highest levels inside the eggs (Fig. 1B, arrows), while they were depleted in the surrounding liver tissue compared to non-infected (ni) controls (Fig. 1B, Fig. S1 and 2). Please note that the color encodes the concentration of the target molecule shown in Fig. 1B and Fig. S1 and 2.

In total, we detected 77 TGs with altered concentrations – 33 were depleted and 43 enriched in livers of bs-infected hamsters compared to the controls (Fig. S3). Adult schistosomes modify fatty acids from their host for biosynthetic purposes and incorporate those in phospholipids and neutral lipids.^35^ Here, we demonstrate that distinct isoforms of dimethyl-phosphatidylethanolamine (DMPE) were enriched in the eggs while they were depleted in the granuloma and in the surrounding liver parenchyma (Fig. 1C). Representative MS images of different lipid classes are shown for the three animal groups (Fig. S4, Fig. 1C focuses on the distribution of the lipids in and around the granuloma). We detected characteristic distributions of lipids in the liver of bs-infected *vs*. ms-infected and ni animals. DMPE (18:2/22:6) was evenly distributed in the parenchyma of bs-infected hamster livers but depleted in granuloma and enriched in eggs (Fig. S4B). On the other hand, DMPE (18:0/22:5) was found in parenchyma and enriched in granuloma and eggs (Fig. S4C). DMPE (18:0/22:4) was not detected in hepatic parenchyma but in granulomas, with enrichment in eggs (Fig. S4D). DMPE (15:0/18:2) was found evenly distributed in all samples apart from the granulomatous regions of bs-infected samples (Fig. S4E). A different lipid species, phosphatidylcholine (PC) (17:2/18:3) was enriched in the outer borders of granulomas but evenly distributed in samples from both controls (Fig. S4F). The distribution of other lipid species, like lysophosphatidylethanolamines, phosphatidylethanolamines (PE), and ceramides are shown in Fig. S5, with an overlay of different lipid species in Figs S6 and S7 (please note, that the colors depict different lipid species in Fig. S6 and S7, while the color encodes the concentration of one target molecule in Fig. 1B and Fig. S1-2 and S4-5). While the DMPE(18:3_20:4) (Fig. S6B and in Fig. S6E in blue) was depleted in the granulomatous area (dashed lines), PC(15:0_22:6) was enriched (in red in Fig. S6C and E, red arrowheads). Granulomas and surrounding tissues were differentiated, and a substructure was detected within the granuloma. Plasmenyl-PE(O-18:0_20:4) (green arrows) was only found in the outer regions of the granulomas. All lipids detected in this region were identified as plasmalogens. Further phosphatidylinositol (18:0_22:4) (Fig. S7A, red arrowheads) was enriched in distinct parts of granulomas while plasmenyl-PE (P-16:0_18:1) (green arrows) was found in the center and around the periphery of the granulomas (Fig. S7).

Next, we quantified spatioregionally occurring lipids by HPTLC–fluorescence detection using the primuline reagent. The chromatogram and the respective densitogram of the non-polar lipids (Fig. S8A,B) showed well-separated blue fluorescence of cholesterol (retention factor [*hR*_F_] 13), oleic acid (*hR*_F_ 22), triolein (*hR*_F_ 45) and cholesteryl oleate (*hR*_F_ 79). A reagent sequence of: first primuline and then phosphomolybdic acid on the same plate resulted in dark green lipid zones (Fig. S9). The tentative assignment of the different lipid species (based on *hR*_F_ and reference substances) was confirmed by HPTLC–HRMS, shown for the liver samples of *S. mansoni* ms- and bs-infected hamsters (Fig. S10, track 5 *vs.* 15). Here, we unambiguously confirmed the presence of oleic acid (*m/z* 281.2486), triolein (*m/z* 907.7727) and cholesteryl oleate (*m/z* 673.5896) (Fig. S10A,B and D–G). Oleic acid, stearic acid (*m/z* 283.2642), linoleic acid (*m/z* 279.2329), and linolenic acid (*m/z* 277.2171) were present in liver samples of ms-infected hamsters (Fig. S10D). Cholesterol (Fig. S10C) showed the mass signals of two oxidation products at *m/z* 425.3391 ([M+O+Na]^+^) and 441.3340 ([M+2O+Na]^+^, and only a very weak signal at *m/z* 409.3443 [M+Na]^+^, all in accordance with the mass signals of liver samples of ms-infected hamsters. In ms-infected hamsters, triolein or similar TGs were absent (Fig. S10A,E). However, we confirmed a strongly fluorescing zone of cholesteryl oleate (Fig. S10A,F,G), which was absent in liver samples of bs-infected hamsters. The comparatively more complex mass spectra of phosphatidic acid, sphingomyelin, PC, and PE were assigned to each respective lipid class (Fig. S11), whose presence we confirmed in the samples, except for phosphatidic acid. The low phosphatidic acid content (Fig. S8B, weakly blue fluorescent zone at *hR*_F_ of 9) was most likely masked by the mass signals of saccharides (glucose) and amino acids (Fig. S9C), whose coelution was proven by derivatization (Fig. S9B, red zone at *hR*_F_ 5 and Fig. S9C, blue-grey zone at *hR*_F_ 6). The quantitative analysis of the eight lipid compounds in the 15 samples (Table S1) showed good reproducibility (mean % relative SD 7.9%, n = 3, range 0−29%). The performance of the linear calibration lines was sufficient (coefficients of determination >0.997 and reproducibilities % relative SD <10%, n = 3). The quantitative results revealed enhanced hepatic levels of sphingomyelins and oleic acid in bs-infected animals, while global hepatic levels of PE, PC, triolein, and cholesterol showed no alteration (Fig. 1D and Figs S8−11).

### *S. mansoni* eggs exhaust hepatocellular neutral lipids

**Fig. 2:**
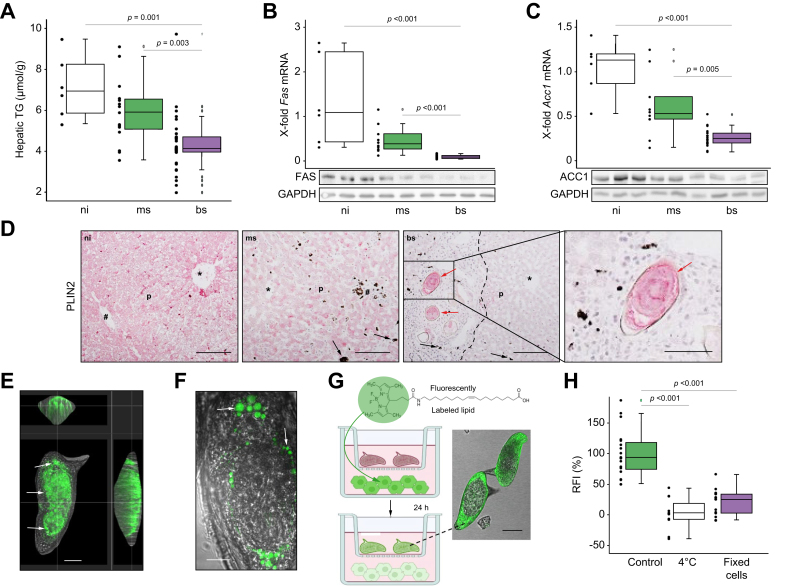
*S. mansoni* infection exhausts the host's hepatic neutral lipid depots while parasite eggs accumulate lipids. (A) Hepatic TG content was reduced in bs-infected hamsters. (B and C) Hepatic mRNA and protein levels of fatty acid synthase (*Fas*) and acetyl-CoA-carboxylase 1 (*Acc1*), both rate-limiting enzymes for lipid synthesis, were reduced in bs-infected hamsters. Representative western blots are depicted. (D) PLIN2-staining visualized the reduction of neutral lipids in liver parenchyma (p) of bs-infected hamsters and the accumulation of neutral lipids in *S. mansoni* eggs (red arrows). Black arrowheads depict *S. mansoni*-infection-specific hepatic hemosiderin deposits, ∗central vein, #portal tract, 200x, bar 100 μm, black dotted line indicates granuloma. (E and F) Confocal microscopy clearly demonstrated the sites of lipid accumulation (white arrows) inside the eggs (liver) cultured with flOA (in green), bars 20 μm (E) and 10 μm (F). A live video showing the active uptake of flOA by an egg (liver) was deposited online (supplementary video 1) and also a 3D video demonstrating the lipid distribution inside liver-extracted eggs can be found online (supplementary video 2). (G) To prove if *S. mansoni* eggs mobilize and take up lipids from hepatocytes, HepG2 cells were fed with flOA, washed three times and subsequently co-cultivated with eggs in a transwell system. Around 3% of the eggs freshly isolated from the host liver took up flOA from HepG2 cells after 24 h of co-culture. The workflow of the experiment depicted schematically (left panel) and representative pictures of a liver-derived egg that took up flOA (bar 25 μm, right panel). Remarkably, nearly 100% of pre-matured *in vitro*-laid eggs took up flOA from HepG2 cells. These experiments were performed at least three times independently. (H) CLSM-based quantification of fluorescence intensity of individual eggs from coculture performed at 4°C (white bar) or with 4% PFA fixed HepG2 cells (purple bar) in comparison to the conventional experiment as a control (con, green bar). At least 10 eggs per condition were analyzed in each of two independent experiments. Levels of significance are indicated in the figure (Kruskal-Wallis-test). ACC, acetyl-CoA carboxylase; bs, bisex; FAS, fatty acid synthase; flOA, fluorescently labeled oleic acid; GAPDH, glyceraldehyde 3-phosphate dehydrogenase; ms, monosex; ni, non-infected; PLIN, perilipin; RFI, relative fluorescence intensity; TG, triglyceride.

Next, we analyzed the concentration of TGs in whole liver lysates of bs-, ms- and ni-hamsters (Fig. 2A). In line with a significant reduction of whole hepatic TGs in bs-samples compared to ms and ni, we detected the corresponding downregulation of hepatic mRNA and protein expression levels of rate-limiting enzymes for lipid synthesis, namely fatty acid synthase and acetyl-CoA-carboxylase, in liver lysates of bs-infected hamsters (Fig. 2B,C). Notably, the differences between ms-infected- and bs-infected hamsters underline that the bisexual infection with egg-production caused pronounced effects (Fig. 2A-C). Immunohistochemistry revealed that infection with *S. mansoni* reduced parenchymal fatty acid synthase expression with the exception of perigranulomatous hepatocytes (red arrow Fig. S12).

### *S. mansoni* eggs mobilize and take up lipids from hepatocytes

Furthermore, we observed a strong reduction of perilipin 2 (PLIN2), a marker for neutral lipid storage, in the liver parenchyma compared to ni- and ms-infected liver specimens (Fig. 2D). Notably, eggs were positively stained for PLIN2 (red arrows, Fig. 2D). The accumulation of neutral lipids in the eggs was confirmed by Oil-Red O staining (red arrowhead, Fig. S13). Please note that some perigranulomatuos hepatocytes were positive on Oil-Red O staining (red arrow, Fig. S13).

Next, we investigated whether the eggs of *S. mansoni* were able to actively take up lipids. We found flOA uptake by freshly isolated eggs using confocal live-cell imaging (supplementary videos 1 and 2) and confocal fluorescence microscopy (Fig. 2E-F and Fig. S14A-C). The uptake occurred rapidly in some living eggs, starting after 10 min (Fig. S14A,B). We also observed flOA in fully developed miracidia (Fig. S14C). Next, we analyzed the uptake of flOA into *in vitro*-laid eggs of different maturity over 18 h (Fig. S15). Eggs incorporated lipids independent of their maturity stage. Depending on their maturity, however, lipids accumulated in distinct structures of the egg. Young eggs (Fig. S15A) incorporated flOA into their vitelline cells. The oocyte remained negative. During embryo development, flOA-positive vitelline-cell content was found inside the embryo as small granular structures as well as in the sub-shell area (Fig. S15B). When the miracidium matured, the sub-shell envelopes (Reynolds‘ layer and van-Lichtenberg‘s envelope) took up flOA (Fig. S15C). Additionally, we questioned whether eggs mobilize and take up lipids from human hepatocytes. As shown schematically in Fig. 2G, we co-cultured *in vitro*-laid eggs with HepG2 cells, which had been pretreated with flOA (Fig. S16) 24 h prior to the coculture. Nearly all pre-matured *in vitro-*laid eggs took up flOA from HepG2 in transwell cocultures (Fig. 2G) but also in direct cocultures (Fig. S14D-E). Based on a previous study design,^36^ we performed two additional control experiments and demonstrated that the fixation of flOA-fed HepG2 cells or co-culturing of flOA-fed HepG2 cells with *in vitro*-laid eggs at 4 °C impaired flOA uptake by the eggs, suggesting that we were measuring a process that requires an active cellular metabolism (Fig. 2H).

Supplementary data to this article can be found online at https://doi.org/10.1016/j.jhepr.2022.100625.

The following are the supplementary data to this article:

Supplementary Video S1

Supplementary Video S2

### *S. mansoni* eggs dysregulate carbohydrate metabolism

**Fig. 3:**
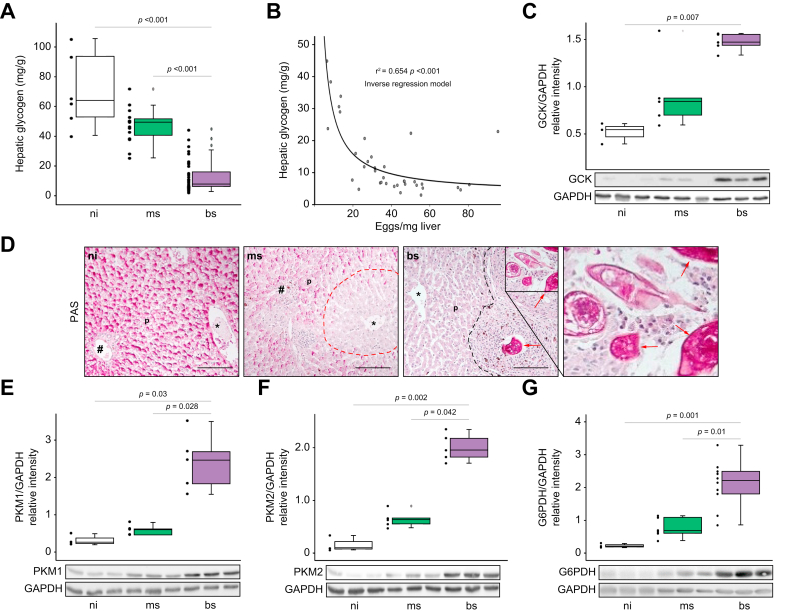
*S. mansoni* infection exhausts the host's hepatic carbohydrate storage. (A) The hepatic glycogen content was diminished approximately 6-fold in livers of bs-infected hamsters. (B) Hepatic glycogen content inversely correlated with the number of eggs per mg of liver tissue. (C) Glucokinase, the first rate-limiting enzyme of glycolysis, was upregulated in livers of bs-infected hamsters. A representative western blot is shown. (D) PAS staining of histologic liver sections revealed homogenous hepatic glycogen storage in ni animals (left panel), zoned glycogen storage in livers of ms-infected hamsters with a reduction of glycogen deposits around the central veins∗ (dotted red line indicates zonation), as well as a complete absence of glycogen in the liver parenchyma (p) of bs-infected hamsters, while eggs were strongly positive stained (red arrows). ∗central vein, #portal tract, 200 x, bar 100 μm, black dotted line indicates granuloma. (E and F) Western blot analysis demonstrated the induction of rate-limiting enzymes of glycolysis like PKM1 (E) and PKM2 (F) in livers of bs-infected hamsters. (G) G6PDH, the rate-limiting enzyme of the PPP of glycolysis, was also upregulated in livers of bs-infected hamsters, n = 3-5. These experiments were performed at least three times independently. Levels of significance are indicated in the figure (Kruskal-Wallis-test and non-linear regression). bs, bisex; flOA, fluorescently labeled oleic acid; G6PDH, Glucose-6-phosphate dehydrogenase; GCK; glucokinase; ms, monosex; ni, non-infected; PAS, periodic acid-Schiff; PKM, pyruvate kinase muscle isozyme; PPP, pentose phosphate pathway.

Next, we analyzed in detail how *S. mansoni* infection affects the hepatic carbohydrate metabolism of the host. As depicted in Fig. 3A, we found hepatic glycogen to be significantly reduced in livers of bs-compared to ms-infected and ni-hamsters. Furthermore, the amount of hepatic glycogen inversely correlated with the number of eggs per mg of hamster liver (Fig. 3B). In line with glycogen exhaustion, we found a significant downregulation of rate-limiting enzymes responsible for glycogen turnover such as glycogen synthase (GS) and glycogen phosphorylase in liver lysates of bs-infected hamsters (Fig. S17). Remarkably, hepatic protein levels of the rate-limiting glycolysis-initiating enzyme glucokinase were significantly increased in the bs group (Fig. 3C). PAS staining visualized a deprivation of intracellular glycogen in parenchymal cells, while the eggs were strongly stained and thus appeared to be rich in glycogen (Fig. 3D). Western blot and immunohistochemistry demonstrated a significant increase in the hepatic protein levels of the glycolytic enzymes pyruvate kinase 1 and 2 (PKM1 and 2), both in bs-infected animals (Fig. 3E,F). Notably, PKM1 and PKM2 occurred in different areas and cells in the livers of bs-infected hamsters, *i.e.* PKM1 in the hepatic parenchyma (Fig. S18A) and PKM2 in the granulomas (Fig. S18B). The opposite regulation of elevated glycolysis and reduced glycogenesis rate-limiting enzymes was confirmed by stimulating human hepatoma cell lines with SEA (Fig. S19). Pyruvate dehydrogenase (PDH) catalyzes the conversion of pyruvate, the end-product of glycolysis, into acetyl-CoA, which links glycolysis and the citric acid cycle. Hepatic PDH was induced by *S. mansoni* infection (Fig. S20). In addition, we observed an elevation of glucose-6-phosphate dehydrogenase, which catalyzes the transition of glucose-6-phosphate into 6-phosphogluconate, the first step in the pentose phosphate pathway (Fig. 3G, Fig. S21).

Finally, we wondered which evolutionary mechanisms might have promoted the ability of eggs to reprogram the host's metabolism in order to utilize the host's lipid and glucose reserves. This question led to the idea that the hepatic effects might have their evolutionary origin in the egg's translocation through the bowel wall into the gut lumen. Remarkably, we found a similar regulation of PKM2, GK2, and PEPCK2 expression in the colon of bs-infected hamsters (Fig. S22A-D).

### *S. mansoni* eggs induce oxidative stress

**Fig. 4:**
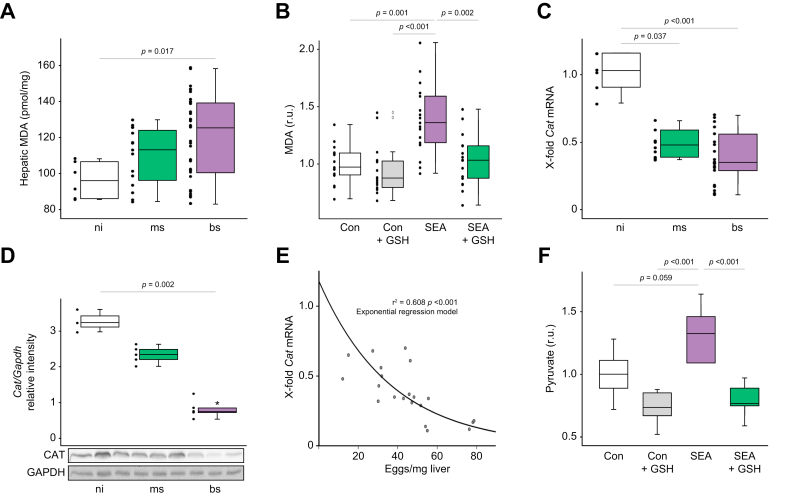
*S. mansoni* egg-induced hepatocellular oxidative stress. (A) Hepatic MDA levels were increased in hamster livers upon bs infection. (B) SEA stimulation induced MDA in HepG2 cells. The induction was abolished by the addition of reduction equivalents in form of GSH (n = 6). (C and D) Hepatic catalase expression is reduced in hamster liver of the bs group (C, mRNA, D, protein level). (E) Hepatic catalase mRNA levels and the number of eggs per mg of liver tissue inversely correlated with exponential trend (regression analysis). Data were normalized to the control group. (F) Pyruvate quantification demonstrated SEA-induced glycolysis in HepG2 cells, which was reduced to unstimulated levels by the addition of reduction equivalents in the form of GSH. The experiment was repeated three times. Data were normalized to the control group. Kruskall-Wallis test was performed to assess group differences. These experiments were performed at least three times independently. Levels of significance are indicated in the figure (Kruskal-Wallis test). bs, bisex; CAT, catalase; Con, control; GSH, reduced L-glutathione; MDA, malondialdehyde; ms, monosex; ni, non-infected; SEA, soluble egg antigens.

Glucose-6-phosphate dehydrogenase is a key regulator of the oxidative branch of the pentose phosphate pathway and is indispensable for maintaining the cytosolic pool of NADPH, thus being essential for the cellular redox balance.^37^ We observed a significant augmentation of MDA, a secondary reaction product of lipid peroxidation, in livers of *S. mansoni*-infected hamsters (Fig. 4A). GSH (reduced L-glutathione) significantly decreased SEA-induced MDA in human hepatoma cells (Fig. 4B). Catalase is one of the crucial antioxidant enzymes that mitigates oxidative stress by destroying cellular hydrogen peroxide to produce water and oxygen. We detected significantly downregulated mRNA and protein levels of catalase in hamster livers (Fig. 4C,D). The downregulated mRNA level of the host's catalase in the bs-group correlated with an increasing number of eggs in the liver (Fig. 4E). Notably, catalase mRNA levels also decreased by stimulation with soluble egg products in human hepatoma cells (Fig. S23).

Surprisingly, the infection with *S. manoni* increased hepatic mRNA levels of Mn^2+^-dependent superoxide dismutase (*MnSod*), while *CuZnSod* mRNA levels decreased (Fig. S24A/B). In the bs group, glutathione peroxidase*,* was not regulated (Fig. S24C). SEA treatment induced the accumulation of pyruvate in HepG2 cells, which was reversed by GSH (Fig. 4F). Strikingly, flOA transfer from flOA-fed HepG2 cells to eggs was reduced in the presence of GSH in co-cultures (Fig. S25). Accordingly, we found a similar regulation of glutathione peroxidase expression in the colon of bs-infected hamsters (Fig. S26).

### *S. mansoni* egg-induced oxidative stress activates the proto-oncogene c-Jun and DNA damage

**Fig. 5:**
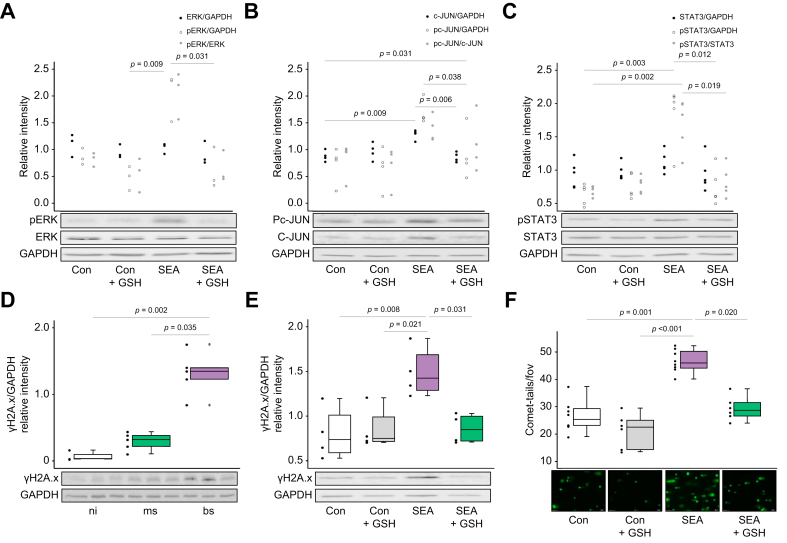
*S. mansoni* eggs induce malignant signaling and oxidative stress-dependent DNA damage in HepG2 cells. (A-C) Western blot analyses revealed the activation of ERK, c-JUN, and STAT3 in SEA-stimulated HepG2 cells. The addition of GSH diminished SEA-induced HCC-associated signaling to control levels. Representative western blots are depicted. (D) γH2A.x, a marker for DNA double strand breaks, is induced in the bs group. (E) Stimulation with SEA induced γH2A.x, while the addition of GSH abolished this effect. (F) Comet assay demonstrated that SEA induced DNA damage in HepG2 cells, and that DNA damage was reduced by addition of GSH. Kruskall-Wallis test was performed to assess group differences. These experiments were performed at least three times independently. Levels of significance are indicated in the figure (Kruskal-Wallis test). bs, bisex; Con, control; γ-H2A.x, phosphorylated form of H2A histone family member X; GSH, reduced L-glutathione; ms, monosex; ni, non-infected; SEA, soluble egg antigens.

We investigated whether the exhaustion of hepatocytes and the associated oxidative stress might trigger the activation of oncogenic signaling and DNA damage. In human hepatoma cells, GSH inhibited the egg product-induced activation of ERK, c-JUN, and STAT3 (Fig. 5A-C). In parallel, we observed significantly elevated protein expression levels of the DNA damage marker γ-H2A.x in bs-infected hamsters and in HepG2 cells following SEA treatment*,* which could be reversed *in vitro* by the ROS scavenger GSH (Fig. 5D,E).

In line with these findings, we demonstrated that SEA induced DNA damage *in vitro* using a comet assay. This effect was reversed by reduced GSH (Fig. 5F). As the antagonization of oxidative stress abolishes SEA-induced c-Jun activation (Fig. 5B), we further analyzed whether the functional activation of AP1 promoter activity is influenced by GSH. The SEA-induced functional activation of AP1 promoter activity was also reduced by GSH in the human colon cell line SW620 (Fig. S27).

### Proof of clinical relevance in human biopsies

**Fig. 6:**
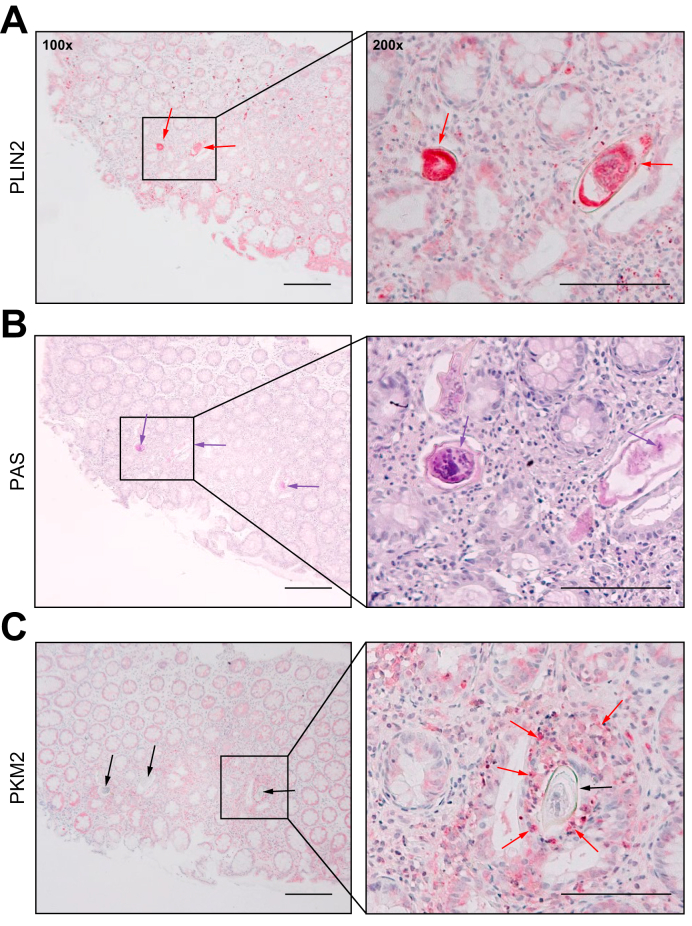
Validation of metabolic implications with tissue samples of a patient infected with *S. mansoni*. (A) Histologic specimen of a colon biopsy from a 23-year-old patient with schistosomiasis was stained for PLIN2. The signal for PLIN2 indicated an accumulation of neutral lipids inside the eggs (red arrows). (B) The positive PAS reaction demonstrated an accumulation of glycogen in most of the eggs passing the bowel wall (purple arrows). (C) Enhanced staining for PKM2 (red arrows) in granulomatous infiltrates around the eggs (black arrows). Representative stainings are depicted, bars 100 (right) and 200 μm (left). PAS, periodic acid-Schiff; PKM, pyruvate kinase muscle isozyme; PLIN, perilipin.

In order to obtain a model-independent proof of the most important results from the hamster and human cell lines, some major targets were visualized in histological slices of colon biopsies that were routinely taken for diagnosis from a 23-year-old male suffering from schistosomiasis (Fig. 6). *S. mansoni* eggs were positively stained for PLIN2 (Fig. 6A) and PAS (Fig. 6B), indicating enhanced accumulation of neutral lipids (A) and glycogen (B) in eggs passing the rectal wall. The staining for PKM2 demonstrated enhanced glycolysis in inflammatory cells around the eggs (Fig. 6C). A visual comparison of PKM2 in infected *vs*. non-infected human tissue is depicted in Fig. S28.

## Discussion

Genes encoding the beta-oxidation pathway are lacking in *S. mansoni* at any stage of its life cycle.^35^ Therefore, *S. mansoni* may not be capable of catabolizing fatty acids for energy production. We demonstrated that eggs mobilized and took-up flOA from well-differentiated human hepatoma cells. The development of larvae requires energy resources but also structural components for the synthesis of cell membranes like lipids that cannot be synthesized by the parasite itself. A single ovum in a newly laid egg does not have high nutritional requirements. After 2-3 days, however, the developing embryo will take more nutrients from its host environment. Moreover, we identified distinct phospholipid species that were enriched in hepatic eggs as previously proposed,^35^ but depleted in granulomas and the surrounding liver parenchyma. Furthermore, lipid mobilization from host cells requires active cellular metabolism and can be suppressed by the addition of a ROS scavenger GSH. During the migration of eggs through host tissues, the uptake of lipids and other molecules might be facilitated by cribriform-like pores inside the eggshell.^38^^,^^39^

Our data strengthen the idea that nutrient uptake from the host is a basic prerequisite for the increase of the eggs' biomass and dimensions during development.^6^^,^^40^ This may explain why we found exhausted hepatic glycogen stores in the presence of eggs. However, the glycogen content of eggs increases with the embryonic development and is highest at the miracidium stage.^40^ It has been observed that *S. mansoni* eggs directly absorb glucose *in vitro*.^35^ Acetyl-CoA and glycerol-3-phosphate, both products of the glycolytic breakdown, serve as components for *de novo* fatty-acid synthesis.^35^ It has been hypothesized that *S. mansoni* eggs store neutral lipids among others for developmental processes of miracidia, which require phospholipids e.g., for synthesizing new membranes.^35^

In *S. japonicum*-infected mice, changes in gene expression of catabolism and anabolism in the liver were affected or could occur via the macrophage M2 phenotype state.^11^ Our data however, clearly demonstrate that soluble egg products modulate the metabolic reprogramming of the host's hepatic carbohydrate and lipid metabolism, independently of any immune response. In line with our findings, maturation of *S. japonicum* eggs depends on cholesteryl ester uptake from HDL and occurs via a CD36-related protein from the egg developmental stages.^41^ Thus, depletion of host lipids/glycogen storage during *S. mansoni* infection is likely effected by the eggs themselves.

Most of the factors we analyzed indicate that the hepatic metabolism of ms-infected hamsters is different to that of bs-infected hamsters, which underlines the role of eggs in these processes. Remarkably, some parameters like PDH or catalase expression are also different between ni- and ms-infected animals, which presumably reflects the effects on hepatic metabolism and oxidative stress that are even caused by adult worms alone. Nevertheless, biomolecular insights into reprogramming of the host’s metabolism by *S. mansoni* might also be important when considering the two sides of the same coin – detrimental malnutrition of infected patients in endemic countries *vs*. beneficial anti-obesity effects that have been explored in the context of obesity.

Metabolic dysregulation as well as DNA damage induced by *S. mansoni* SEA was restored by the addition of a ROS scavenger *in vitro*. Using *in vitro* assays, we demonstrated that SEA is capable of inducing oxidative stress, subsequently resulting in the activation of oncogenic signaling and DNA damage. In addition, we found a metabolism-associated dysregulation of the antioxidant system, which is in line with previous observations.^14^

The phenomenon of exploiting the host's energy reserves constitutes a parasitic principle. In the case of schistosome eggs, however, for the first time we describe metabolic deprivation and effects on carbohydrate as well as lipid regulation. Nutrient deficiency resulted in oxidative stress in the livers of bs-infected hamsters as well as in SEA-stimulated human hepatoma cells *in vitro*. SEA significantly inhibited the expression of catalase *in vitro*, which inversely correlated with elevated egg load *in vivo*. Furthermore, we demonstrated that oncogenic signaling and DNA damage are consequences of egg-mediated metabolic deprivation, and that the ensuing oxidative stress can be neutralized by the addition of the ROS scavenger GSH. Our data suggest that *S. mansoni* leads to metabolic reprogramming of the host, in order to acquire the hosts’ lipid and carbohydrate reserves, absolutely essential for miracidial development within the egg.

In summary, our results shed new light on the schistosome parasite–host parenchyma interaction, at the level of the egg stage. We demonstrate that *S. mansoni* eggs take advantage of the host environment through metabolic reprogramming of hepatocytes and enterocytes. Eggs that end up in the liver cause oxidative stress-induced- and malignancy-associated signaling, as well as DNA damage, which in combination might precondition or promote hepatocellular damage.

## Financial support

This work was supported by grants from Deutsche Forschungsgemeinschaft DFG RO3714/4-1 and Sp314-13-1, INST 162/500-1 FUGG, INST 162/471-1 FUGG; INST 162/536-1 FUGG, GILEAD (support program Infectiology 2017), from Hessian Ministry of Science, Higher Education and Art (HMWK), LOEWE Center DRUID, in part by the Wellcome Trust [CGG, TQ; FUGI, Grant number 107475/Z/15/Z], and the 10.13039/501100009560University Hospital Giessen and Marburg (UKGM).

## Authors’ contributions

MR and ER conceived the project and directed the study. V.vB., M.R., C.G.G., R.A.W., and E.R. were involved in writing of the manuscript. V.vB., L.H., A.B., N.B., S.G., V.W., L.R., S.W., K.T., T.Q., S.H., P.K., S.G., K.W., A.M., G.S., and M.R. performed experiments. T.Q., S.H., B.S., G.S., F.H.F., G.M., K.B., P.W., and C.G.G. supervised experiments and contributed samples, materials, methods and instrumentation. A.B., L.H., S.G., V.W., L.R., S.W., A.M., J.P.-K., and M.R. were involved in statistical analysis. All authors analyzed, interpreted, discussed the data, and reviewed the manuscript.

Please refer to the accompanying ICMJE disclosure forms for further details.

## Data availability statement

All relevant data is contained within the manuscript and supporting information. Raw data are available upon request.

## Conflict of interest

B.S. and C.G.G. are consultants of TransMIT GmbH, Giessen, Germany. The other authors declare that they have no conflicts of interest.

Please refer to the accompanying ICMJE disclosure forms for further details.
