# Supplementary material for: Metabolic reprogramming of hepatocytes by Schistosoma mansoni eggs
Source: JHEP Rep. 2022 Nov 7;5(2):100625. doi: 10.1016/j.jhepr.2022.100625 (PMC9800334; doi:10.1016/j.jhepr.2022.100625)
Supplement: Multimedia component 1 [file mmc1.docx]

**Supplementary Materials**

**Metabolic reprogramming of hepatocytes by *Schistosoma mansoni* eggs**

Verena von Bülow^1^, Sarah Gindner^1^, Anne Baier^1^, Laura Hehr^1†^, Nicola Buss^1^, Lena Russ^1^, Sarah Wrobel^1^, Victoria Wirth^1^, Kuscha Tabatabai^1^, Thomas Quack^2^, Simone Haeberlein^2^, Patrik Kadesch^3^, Stefanie Gerbig^3^, Katja R. Wiedemann^3^, Bernhard Spengler^3^, Annabel Mehl^4^, Gertrud Morlock^4^, Gabriele Schramm^5^, Jörn Pons-Kühnemann^6^, Franco H. Falcone^2^, R. Alan Wilson^7^, Katrin Bankov^8^, Peter Wild^8^, Christoph G. Grevelding^2^, Elke Roeb^1#^, Martin Roderfeld^1#*^

**Table of contents:**

**P3:** Detailed description of MALDI MSI analysis and identification by LC-MS/MS

**P4: SFig. 1:** *S. mansoni* infection caused TAG accumulation in eggs and surrounding granulomas

**P5:** **SFig. 2:** *S. mansoni* infection caused TAG accumulation in eggs and surrounding granulomas (arrowheads) and depletion in the non-affected tissue.

**P6:** **SFig. 3:** Numbers of enriched or depleted triglyceride signals, determined as markers for infection.

**P7:** **SFig. 4:** Distribution of lipid species differs characteristically in bisex-, monosex-infected, and control samples.

**P8:** **SFig. 5:** Distributions of lipid species differ characteristically in bisex-, monosex-infected, and control samples.

**P9:** **SFig. 6:** Individual MSI-scans of distinct lipids and overlay.

**P10:** **SFig. 7:** *S. mansoni* infection caused an altered distribution of hepatic lipids.

**p11:** **SFig. 8:** Quantification of non-polar and polar lipids in liver samples by HPTLC-FLD.

**p12:** **SFig. 9:** Further characterization and detection of liver samples by HPTLC-Vis.

**p13:** **SFig. 10:** Confirmation of assigned non-polar lipids in liver samples by HPTLC-HRMS.

**p14:** **SFig. 11:** Confirmation of assigned polar lipids in liver samples by HPTLC-HRMS.

**p15:** **SFig. 12:** *S. mansoni* infection reduced parenchymal FAS expression with the exception of perigranulomatous hepatocytes.

**p16:** **SFig. 13:** *S. mansoni* infection reduced the accumulation of neutral lipids in parenchyma with the exception of perigranulomatous hepatocytes.

**p17:** **SFig. 14:** Confocal microscopy revealed the uptake and intracellular storage of TopFluor OA (green) into 2-3% of liver derived *S. mansoni* eggs and nearly 100% in pre-matured *in vitro* laid eggs.

**p18: SFig. 15:** Uptake of flOA into *in vitro*-laid eggs of different maturity.

**p19:** **SFig. 16:** Fluorescence microscopy revealed uptake and intracellular storage of TopFluor OA (green) into HepG2 cells.

**P20:** **SFig. 17:** *S. mansoni* bisex infection reduced hepatic glycogen turnover.

**P21:** **SFig. 18:** Enhanced hepatic expression pattern of PKM1 and PKM2 in *S. mansoni* infected hamsters.

**p22:** **SFig. 19:** Soluble egg antigen (SEA) induced glycolysis and reduced glycogen synthesis in HepG2 cells.

**p23:** **SFig. 20:** *S. mansoni* bisex infection induced hepatic levels of pyruvate dehydrogenase.

**p24:** **SFig. 21:** Enhanced hepatic expression pattern of G6PDH in *S. mansoni* infected hamster liver.

**p25:** **SFig. 22:** *S. mansoni* bisex infection modulated the expression of colonic PKM2, PCK2, and GK2.

**p26:** **SFig. 23:** SEA stimulation decreased *catalase* mRNA level in HepG2 cells.

**p27:** **SFig. 24:** *S. mansoni* bisex infection modulated hepatic mRNA of oxidative stress markers.

**p28:** **SFig. 25:** GSH decreased flOA uptake of the eggs from flOA-fed HepG2 cells in coculture.

**p29:** **SFig. 26:** *S. mansoni* bisex infection modulated the expression of colonic *Gsh-Px*.

**P30:** **SFig. 27:** SEA activated promotor activity was reduced by GSH.

**P31: SFig. 28:** Visual comparison of PKM2 in infected vs non-infected tissue.

**P32-34:** Supplementary CTAT Tables

**P35: STable 1:** Quantification results and reproducibilities determined via HPTLC-FLD.

**Detailed description of MALDI MSI analysis and identification by LC-MS/MS**

For matrix-assisted laser desorption/ionization mass spectrometry imaging (MALDI MSI), an ultrafine pneumatic sprayer (SMALDIPrep, TransMIT, Giessen, Germany) was used to apply matrix on cryosections of 20 µm thickness. 2,5-dihydroxybenzoic acid (DHB) (30 mg/mL in H_2_O/acetone (1:1, V/V) with 0.1 vol-% trifluoroacetic acid in the total volume, 100 µL, 10 µL/min) and 1,5-diaminonaphthalene (DAN) (3.3 mg/mL in H_2_O/Methanol (1:9, V/V), 400 µL, 30 µL/min) were used as matrices for positive- and negative-ion mode, respectively. A high-resolution atmospheric-pressure MALDI imaging ion source (AP-SMALDI5 AF, TransMIT) was coupled to a Q Exactive HF orbital trapping mass spectrometer (Thermo Fisher Scientific).

Lipid extractions of liver homogenates were performed for each sample group as described by [Garikapati et al., in revision Analytical and Bioanalytical Chemistry]. For LC-MS/MS, a reversed-phase 1.8 µm column (100 X 2.1 mm, ACQUITY UPLC HSS T3, Waters, Eschborn, Germany) on a UHPLC system (Ultimate 3000 UHPLC, Thermo Fisher Scientific, Dreieich, Germany) was coupled to a Q Exactive HF-X orbital trapping mass spectrometer (Thermo Fisher Scientific, Bremen, Germany). LC gradient and MS instrument settings are described in detail elsewhere [Wiedemann et al., in revision Analytical and Bioanalytical Chemistry].

For annotation of lipid species, ”Lipid Match Flow” was used and statistical analysis was performed using ”Perseus”. Comparing bs-infected and ms-infected, respectively, with ni control samples, ANOVA tests with permutation-based false discovery rate followed by posthoc tests were performed, in order to determine infection markers. Resulting marker lists were further used to generate ion images of the MALDI MSI data using the software ”Mirion”.

**Suppl. Figure 1**


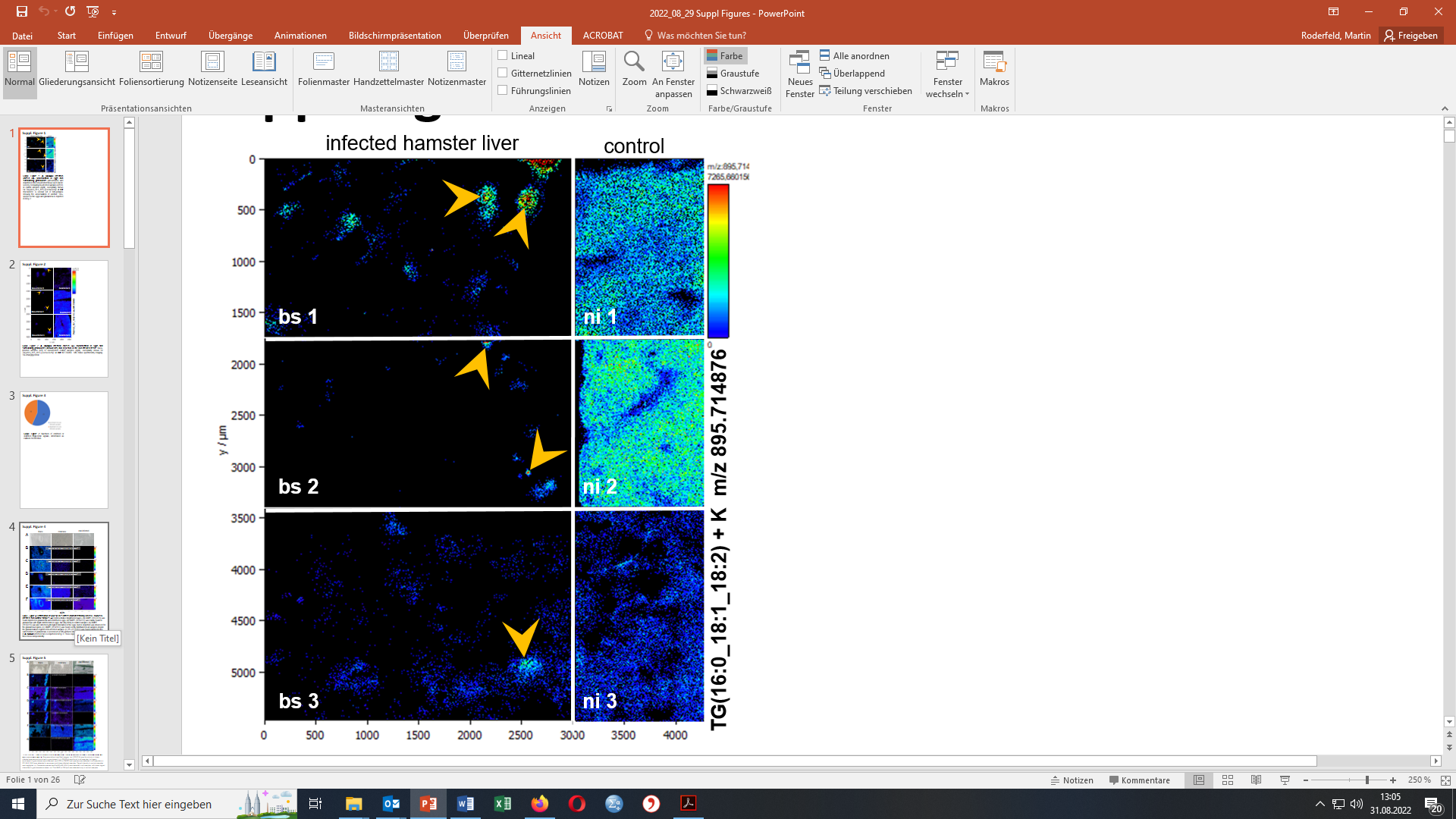


**Suppl. Figure 1: *S. mansoni* infection caused TAG accumulation in eggs and surrounding granulomas** (arrowheads) and depletion in the non-affected tissue as it can be seen by comparing bs-infected samples (left) to ni control samples (right), exemplarily shown for TAG(16:0_16:1_18:1) [C_53_H_98_O_6_+K]^+^ at *m*/*z* 869.699079. A second set of MSI-pictures showing the accumulation of another TAG-species in the eggs and granuloma is depicted in SFig. 2.

**Suppl. Figure 2**


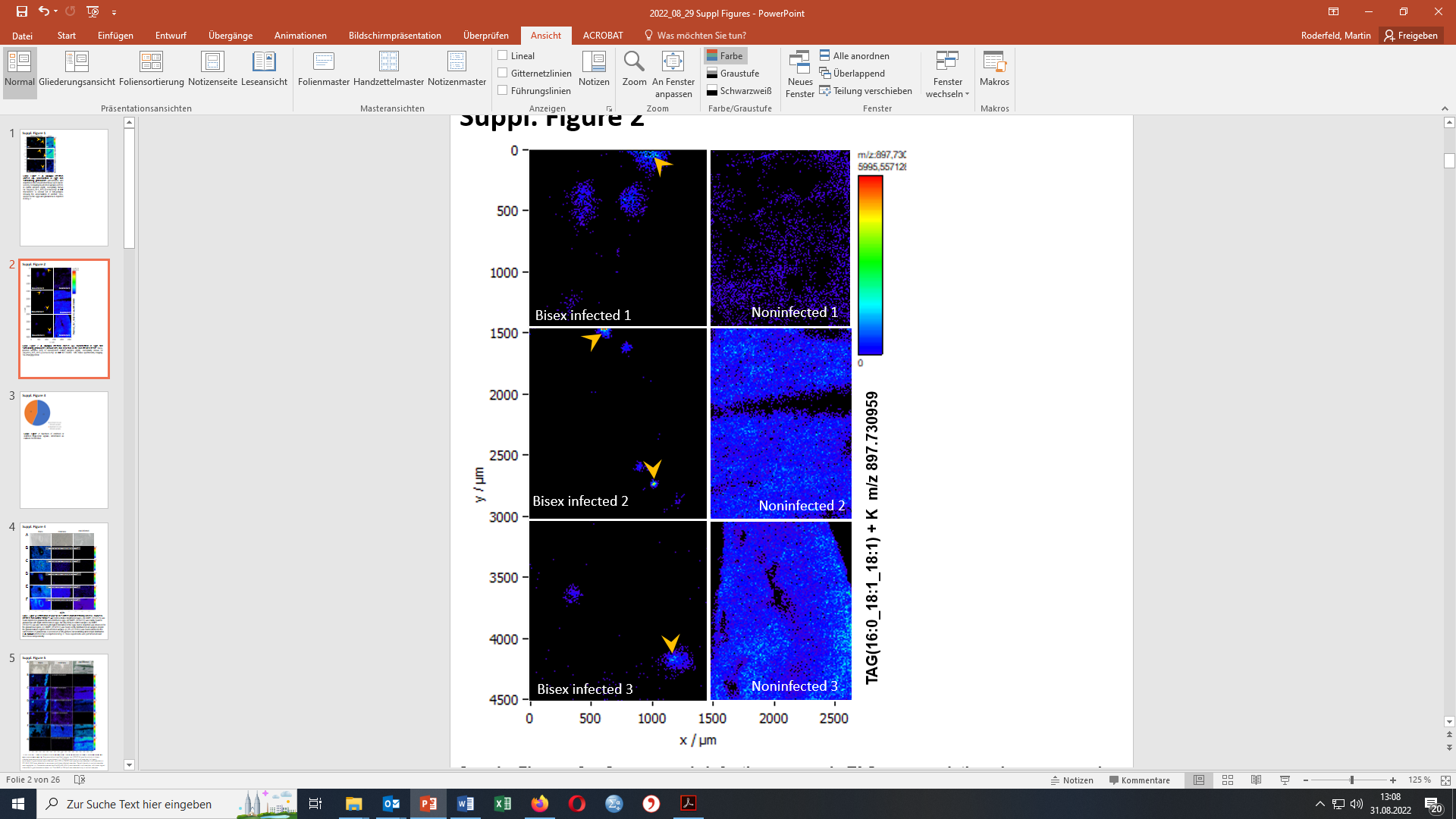


**Suppl. Figure 2: *S. mansoni* infection caused TAG accumulation in eggs and surrounding granulomas** **(arrowheads) and depletion in the non-affected tissue.** Bisex-infected samples (left) to non-infected control samples (right), exemplarily shown for TAG(16:0_18:1_18:1) [C_55_H_102_O_6_+K]^+^ at *m/z* 897.730959. MSI Mass spectrometry imaging, TAG triacylglyceride.

**Suppl. Figure 3**


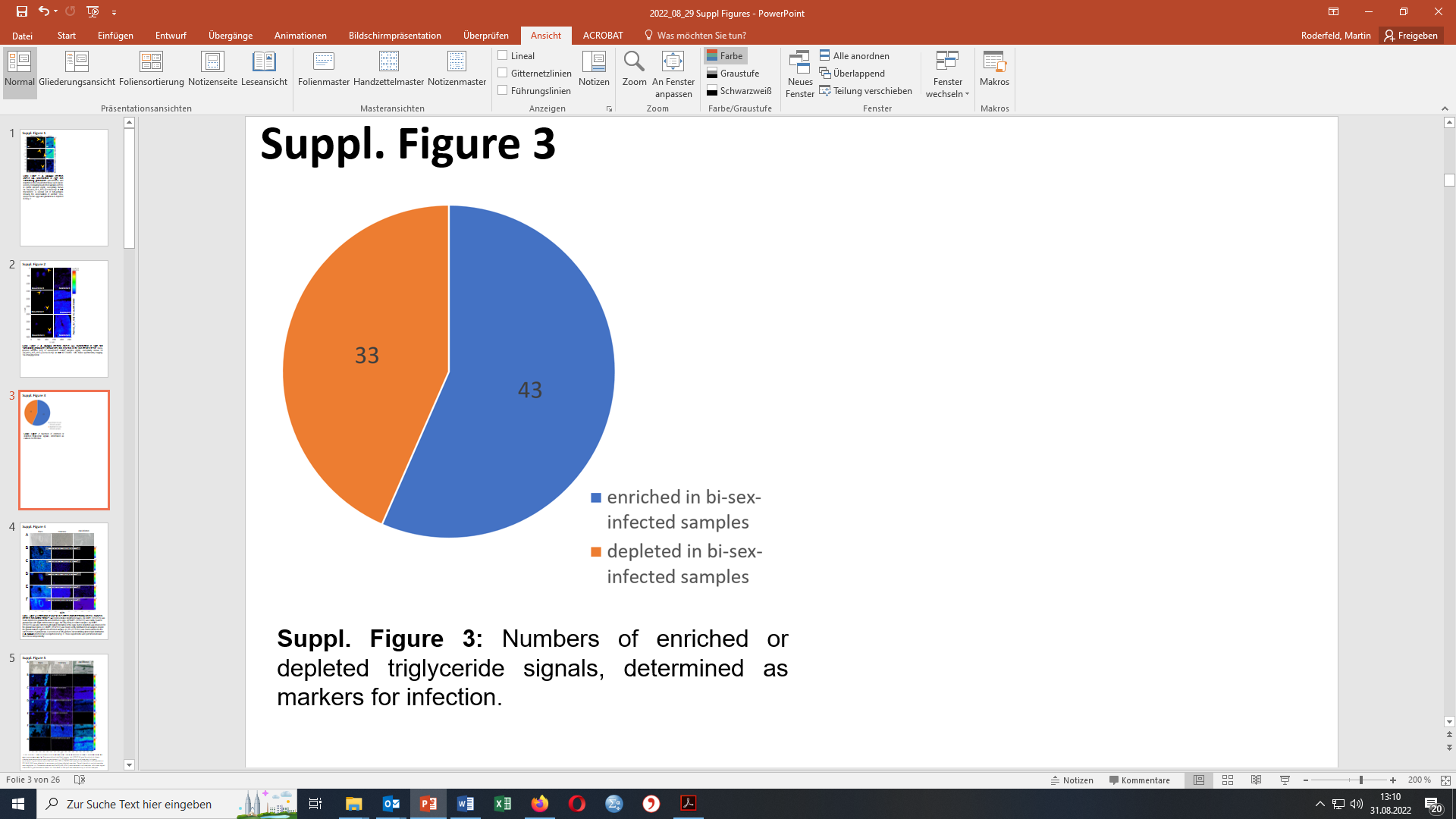


**Suppl. Figure 3: Numbers of enriched or depleted triglyceride signals, determined as markers for infection.**

**Suppl. Figure 4**


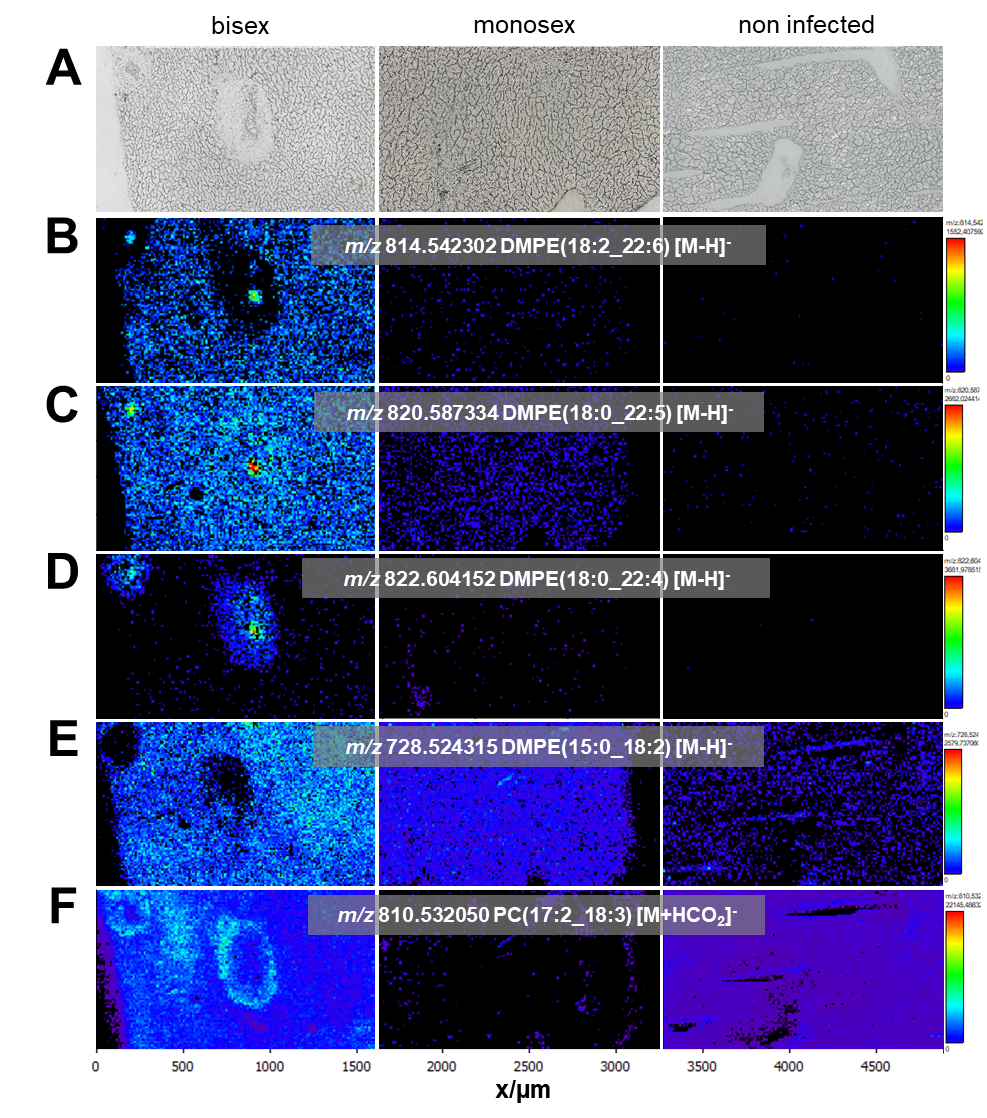


**Suppl. Figure 4: Distribution of lipid species differs characteristically in bisex-, monosex-infected, and control samples.** (**A**) Representative brightfield images. (**B**) DMPE (18:2/22:6) was found depleted in granulomas and enriched in eggs. (**D**) DMPE (18:0/22:4) was mainly found in granulomas with slight enrichments in eggs, but only rarely in control samples. (**C**) DMPE (18:0/22:5) was also detected with higher intensities in the eggs, but no depletion was observed for the granuloma regions. (**E**) DMPE (15:0/18:2) was found evenly distributed in all samples despite the granulomatous regions in bs-infected samples. (**F**) PC (17:2/18:3) was found enriched in the outer borders of granulomas. A second set of MSI-pictures demonstrating altered lipid distribution in *S. mansoni*-infected mice is depicted in SFig. 5. These experiments were performed at least three times independently.

**Suppl. Figure 5**


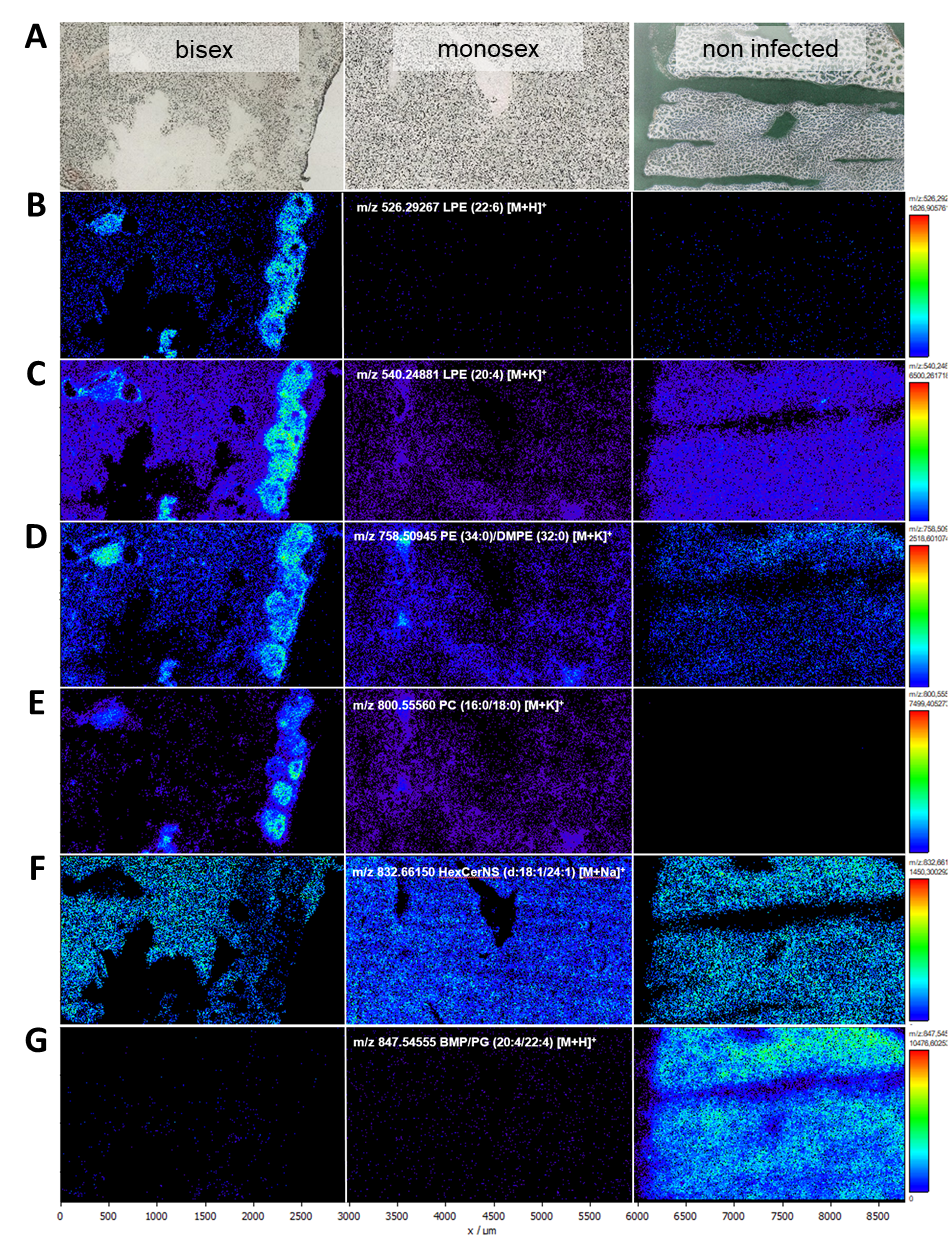


**Suppl. Figure 5: Distributions of lipid species differ characteristically in bisex-, monosex-infected, and control samples.** (**A**) Representative brightfield images. (**B**) LPE(22:6) was found only in bisex-infected samples and enriched in granulomas. (**C**) LPE(20:4) was found in all samples, but again, enrichment in granulomas was observed. (**D**) A PE or DMPE lipid species was detected in all samples (**E**) PC(16:0/ 18:0) was detected in monosex- and bisex-infected samples. Signal intensity in control samples was negligible. (**F**) Ceramide species HexCerNS(d18:1/24:1) was detected in all samples, with lower signal intensities in granulomatous areas. **(G)** This BMP or PG lipid was detected only in control samples.

**Suppl. Figure 6**


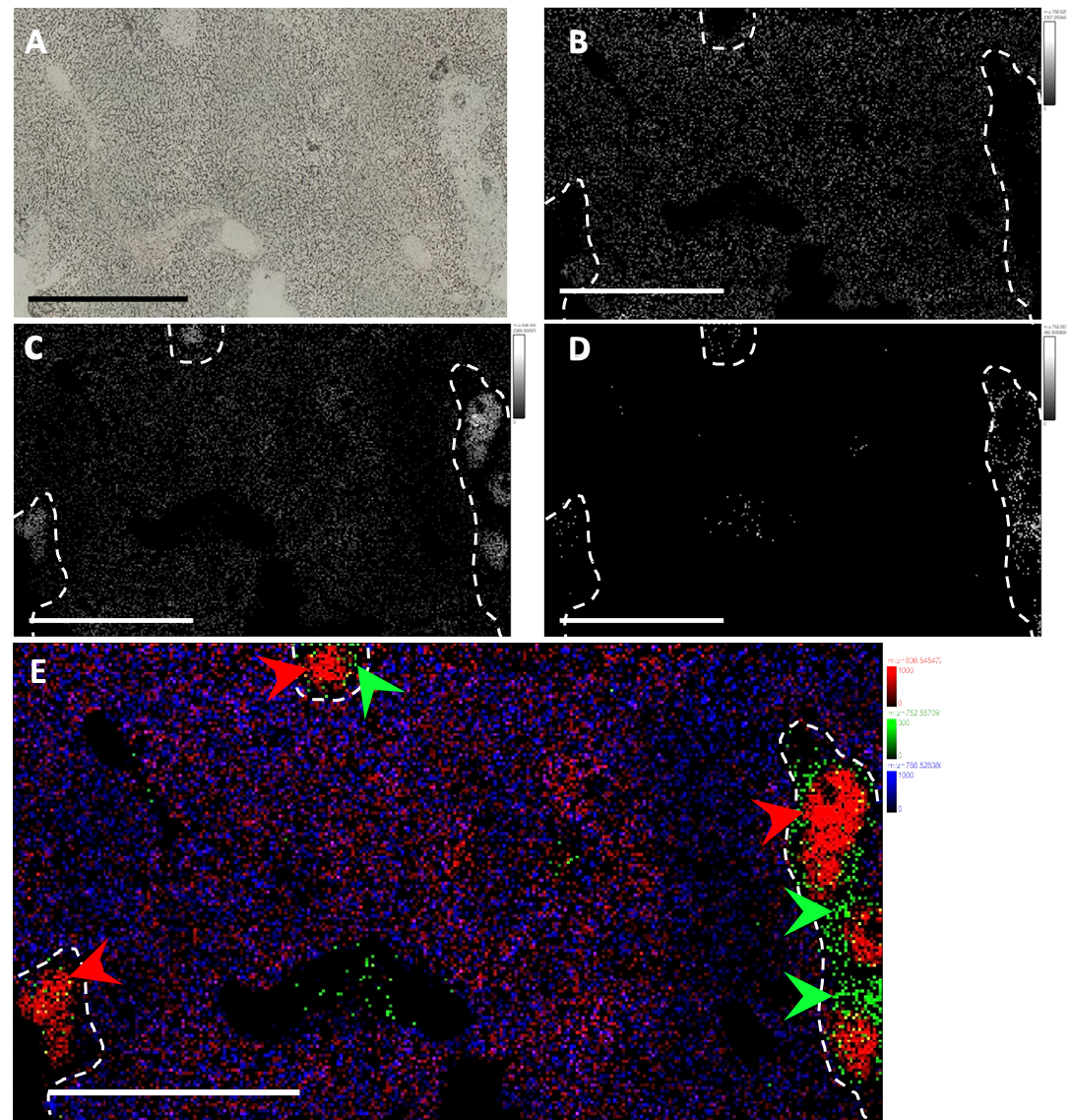


**Suppl. Figure 6: Individual MSI-scans of distinct lipids and overlay.** **(A)** Representative brightfield picture of the area of interest in the liver of a bisex-infected hamster. **(B)-(D)** Representative MSI scans at 10 µm pixel size visualize altered ion distributions of (B) *m*/*z* 788.528381, identified as N,N-dimethyl phosphatidylethanolamine DMPE(18:3_20:4) [C_45_H_76_NO_8_P−H]^−^, **(C)** *m*/*z* 836.545472, identified as phosphatidylcholine PC(15:0_22:6) [C_45_H_78_NO_8_P+HCO_2_]^−^, and **(D)** *m*/*z* 752.557091, identified as phosphatidylethanolamine plasmenyl-PE(O-18:0_20:4) [C_43_H_80_NO_7_P −H]^−^. Dashed lines indicate granulomas. Bars 500µm. **(E)** Overlay of individual MSI scans for DMPE(18:3_20:4) (blue), PC(15:0_22:6) (red), and plasmenyl-PE(O-18:0_20:4) (green). A second example of a representative MSI-scan focussing on a granuloma is depicted in SFig. 2.

**Suppl. Figure 7**


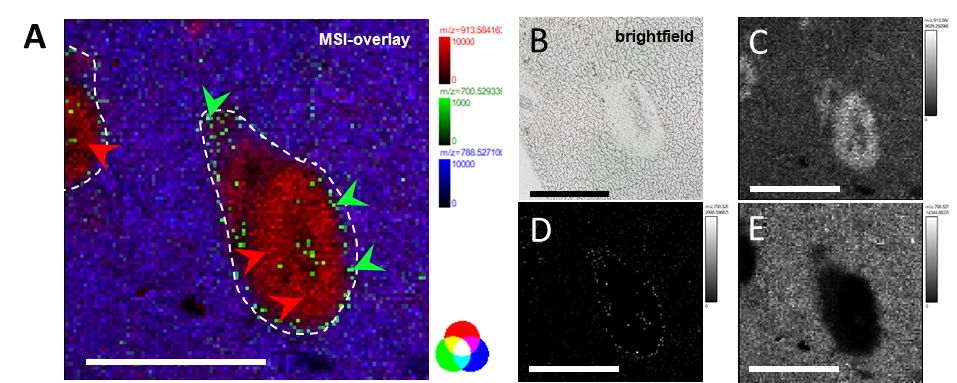


**Suppl. Figure 7: *S. mansoni* infection caused an altered distribution of hepatic lipids**. MSI overlay **(A)**, brightfield **(B)**, and individual MSI-scans **(C-E)** of distinct lipids. **(B)** Representative brightfield picture of the area of interest in the liver of a bisex-infected hamster. **(C)-(E)** MSI scans of ions representing distinct lipids **(C)** *m/z* 913.584163 (red) PI(18:0_22:4), [M−H]^−^ **(D)** *m/z* 700.529339 (green) plasmenyl-PE(P-16:0/18:1) [M−H]- **(E)** *m/z* 788.527100 (blue) DMPE(18:3_20:4), [M−H]^−^. Dashed lines indicate granulomas. Scale bars 500 µm, MSI Mass spectrometry imaging, DMPE dimethyl-phosphatidylethanolamine, PE phosphatidylethanolamine, PI phosphatidylinositol.

**Suppl. Figure 8**


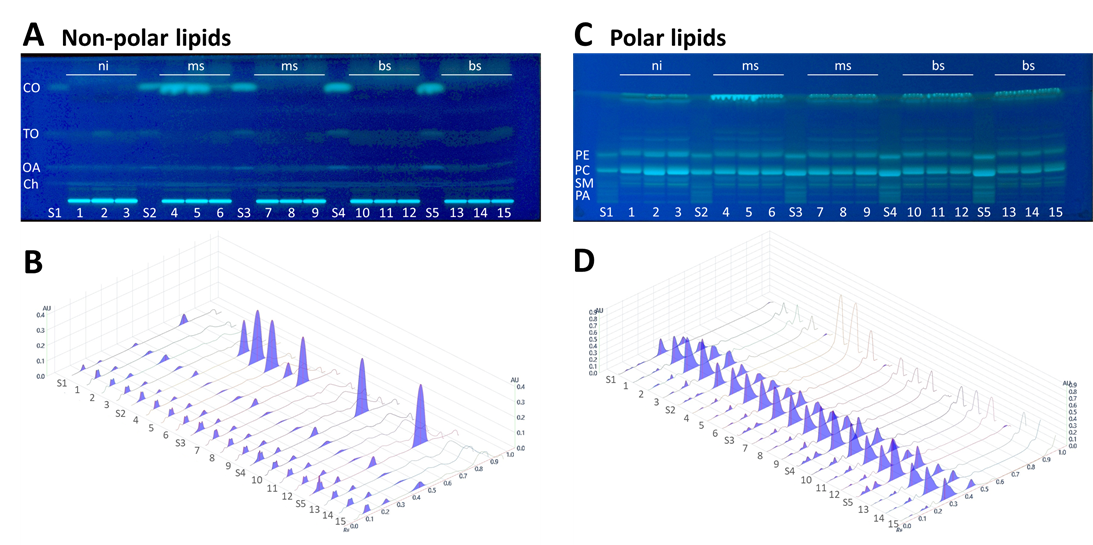


**Suppl. Figure 8: Quantification of non-polar and polar lipids in liver samples by HPTLC-FLD.** Chromatograms **(A/C)** and corresponding 3D densitograms **(B/D)** of sample tracks no. 1−15 (ni = non-infected, ms = monosex-infected, bs = bisex-infected hamsters) and five calibration standard levels (S1−S5) of non-polar lipids (A/B: Ch= cholesterol, OA = oleic acid, TO = triolein, CO = cholesteryl oleate) and polar lipids (C/D; PA = phosphatidic acid, SM = sphingomyeline, PC = phosphatidylcholine, PE = phosphatidylethanolamine).

**Suppl. Figure 9**


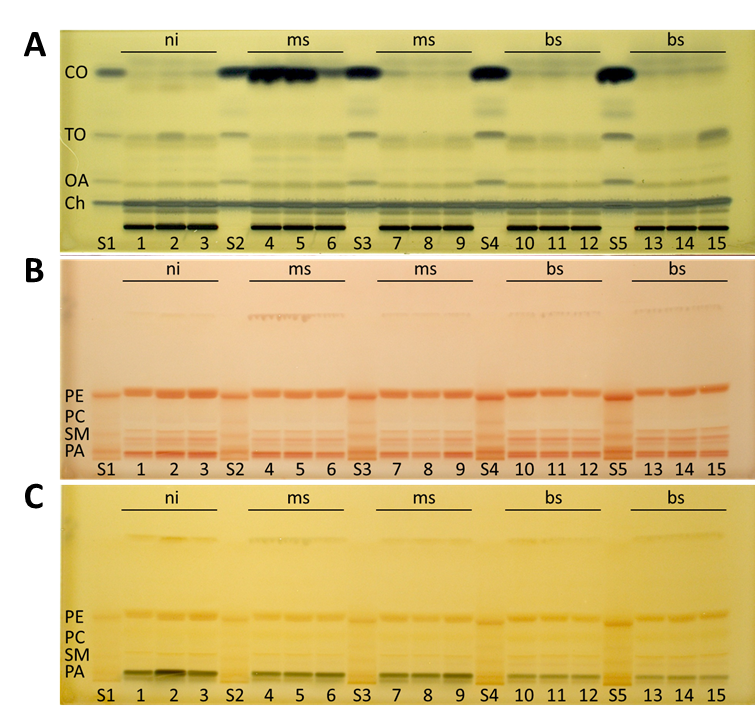


**Suppl. Figure 9: Further characterization and detection of liver samples by HPTLC-Vis.** Chromatograms of non-polar **(A**; Ch = cholesterol, OA = oleic acid, TO = trioleine, CO = cholesteryl oleate) and polar lipids **(B,C**; PA = phosphatidic acid, SM = sphingomyeline, PC = phosphatidylcholine, PE = phosphatidylethanolamine) in sample tracks no. 1−15 (ni = non-infected, ms = monosex-infected, bs = bisex-infected hamsters) and five standard calibration levels (S1−S5). As reagent sequence, the primuline-treated plate was derivatized with phosphomolybdic acid **(A)**, or for detection of amino acids or derivatives like PE and saccharides (glucose), with ninhydrin **(B)** and diphenylamine-aniline o-phosporic acid reagents **(C)**, respectively.

**Suppl. Figure 10**


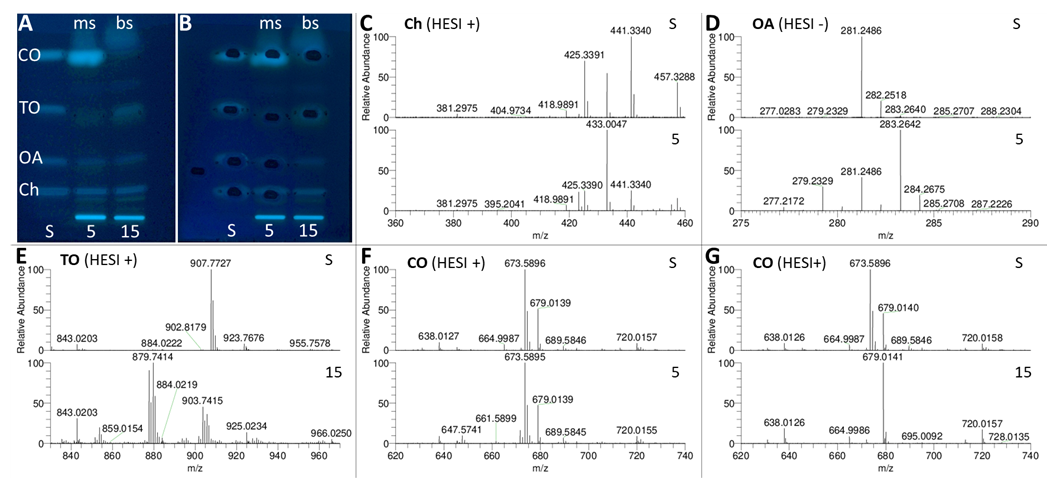


**Suppl. Figure 10: Confirmation of assigned non-polar lipids in liver samples by HPTLC-HRMS.** HPTLC-FLD chromatograms before **(A)** and after **(B)** online elution of the zones of interest via TLC-MS Interface 2 into the HRMS. Mass spectra **(C-G)** of standard zones (S) and respective sample zones (ms = monosex-infected hamster on track no. 5 and bs = bisex-infected hamster on track no. 15).

**Suppl. Figure 11**


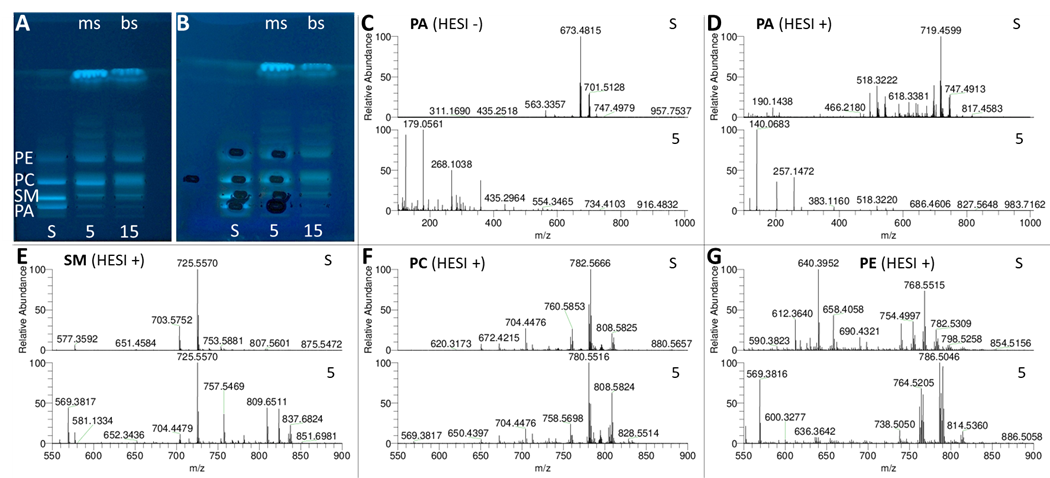


**Suppl. Figure 11: Confirmation of assigned polar lipids in liver samples by HPTLC-HRMS.** HPTLC-FLD chromatograms before **(A)** and after **(B)** online elution of the zones of interest via TLC-MS Interface 2 into the HRMS. Mass spectra **(C-G)** of standard zones (S) and respective sample zones (ms = monosex-infected hamster on track no. 5 and bs = bisex-infected hamster on track no. 15).

**Suppl. Figure 12**


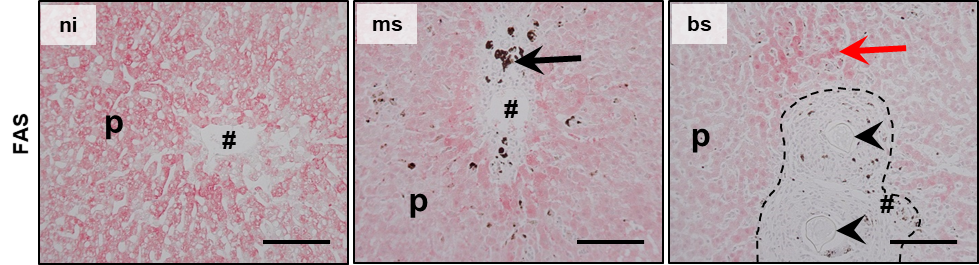


**Suppl. Figure 12: *S. mansoni* infection reduced parenchymal FAS expression with the exception of perigranulomatous hepatocytes.** Fatty acid synthase (FAS) immunostaining (red) in the liver of non-infected (ni), monosex-infected (ms), and bisex-infected (bs) hamsters. Bar 100 µm, p parenchyma, # portal field, ----- granuloma, arrowhead *S. mansoni* eggs, black arrow hemosiderin deposits, red arrow FAS positive perigranulomatous hepatocytes. Representative immunostainings are shown.

**Suppl. Figure 13**


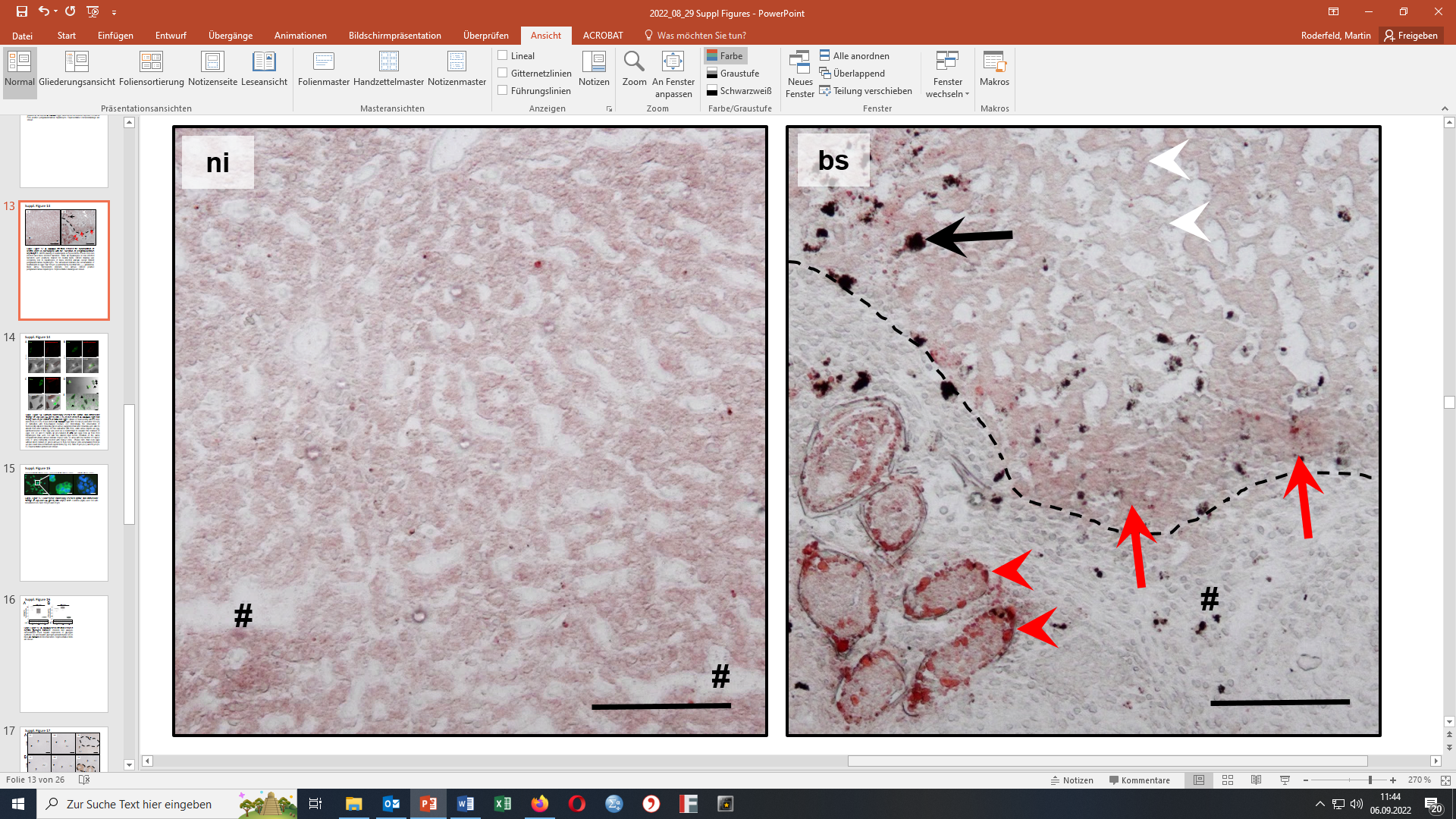


**Suppl. Figure 13: *S. mansoni* infection reduced the accumulation of neutral lipids in parenchyma with the exception of perigranulomatous hepatocytes.** OilRed staining of neutral lipids on cryosections of livers from non infected and bisex infected hamsters. While all hepatocytes in non infected hamsters were positively stained for neutral lipids (left panel), OilRed staining was completely lost in hepatocytes (white arrowheads) of bisex infected animals except distinct perigranulomatous hepatocytes (red arrows). The arrowhead indicates the accumulation of neutral lipids in eggs, white arrowheads: non stained hepatocytes, red arrows: OilRed positive perigranulomatous hepatocytes. Bar 100 µm, p parenchyma, # portal field, ----- granuloma, black arrow hemosiderin deposits. Representative stainings are shown.

**Suppl. Figure 14**


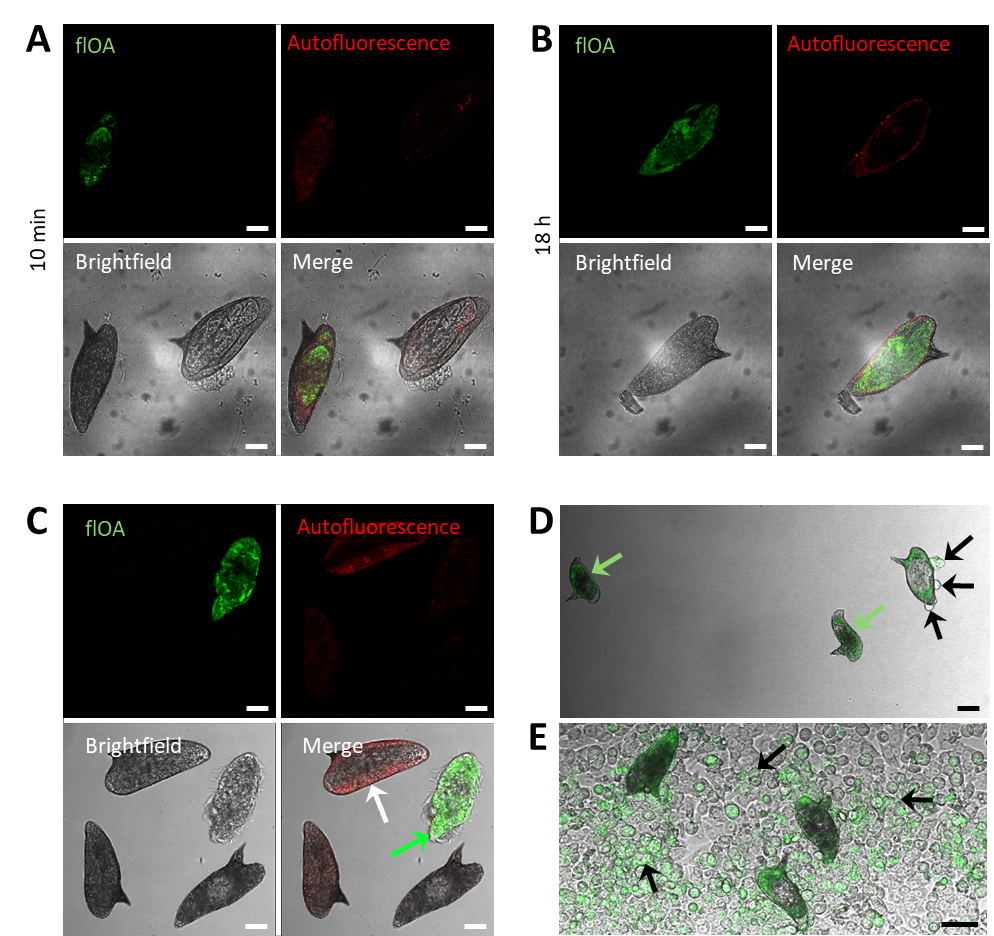


**Suppl. Figure 14: Confocal microscopy revealed the uptake and intracellular storage of TopFluor OA (green) into 2-3% of liver derived *S. mansoni* eggs and nearly 100% in pre-matured *in vitro* laid eggs.** Uptake of fluorescently labeled oleic acid (flOA) in 2-3% of liver-derived *S. mansoni* eggs after 10 min **(A)** and after 18 h **(B)** of cultivation with flOA-charged medium. **(C)** Interestingly, the observation of fluorescently labeled miracidia (green arrow) suggested that also miracidia were able to absorb flOA after hatching. 60 min cultivation with flOA, white arrow depicts an egg, autofluorescence of the egg was used as a counterstain to visualize the contours of eggs: red. **(D and E)** Nearly all pre-matured *in vitro* laid eggs took up flOA from hepatocytes that were fed with the labeled lipid before coculture in the same compartment (black arrows indicate HepG2 cells, D: area with low number of HepG2 cells, E: area confluently covered with HepG2 cells). Please note, that even eggs without direct contact (D, green arrows) to flOA fed HepG2 cells accumulated flOA as we also could show in transwell experiments (Fig. 3H). Bars 25 µm (A-C) and 50 µm (D-E). Representative pictures are shown.

**Suppl. Figure 15**


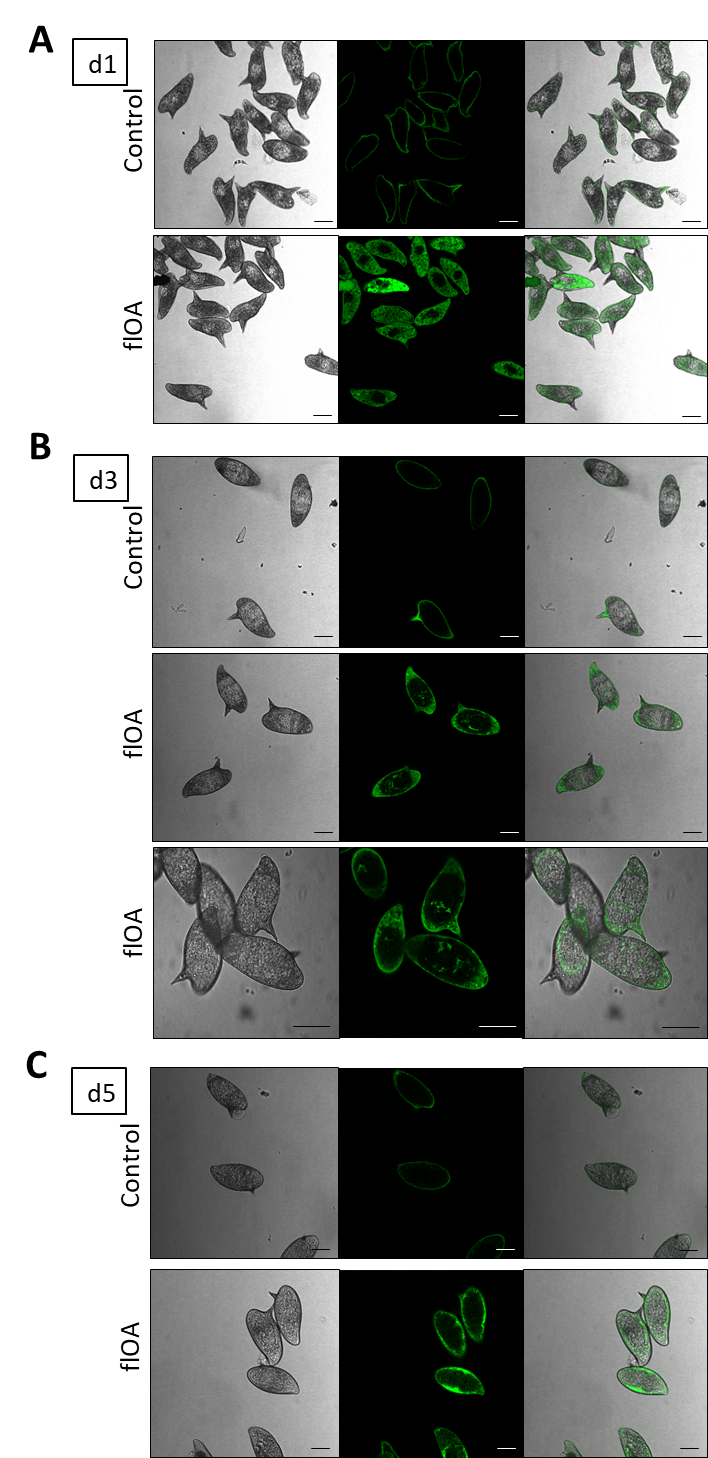


**Suppl. Fig. 15: Uptake of flOA into *in vitro*-laid eggs of different maturity.** Prior to addition of flOA, eggs matured *in vitro* for (A) 1 day, (B) 3 days, (C) 5 days. At these different time points of egg maturation (Jurberg et al. 2009 doi: 10.1007/s00427-009-0285-9), flOA was added, and ist uptake monitored 18 h later. Untreated eggs served as control and showed autofluorescence of the egg shell only. Autofluorescence and flOA fluorescence were excited at 488 nm and recorded using the identical detection window. Eggs in the images are representative for 30-50 eggs per condition. Scale bars: 50 µm.

**Suppl. Figure 16**


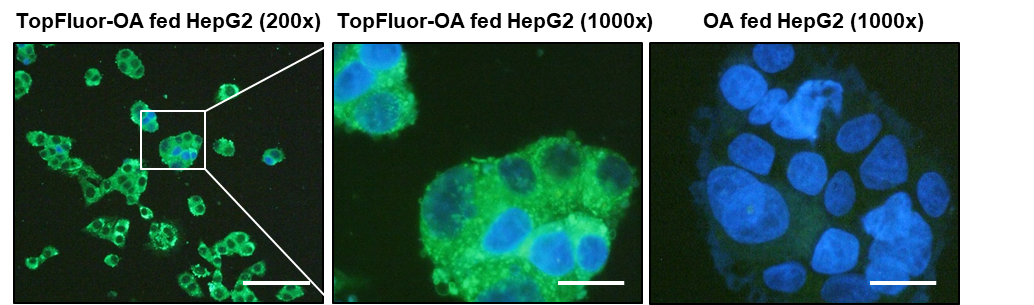


**Suppl. Figure 16: Fluorescence microscopy revealed uptake and intracellular storage of TopFluor OA (green) into HepG2 cells.** Controls (right) were fed with non-labeled OA. Bars 100 µm and 20 µm.

**Suppl. Figure 17**


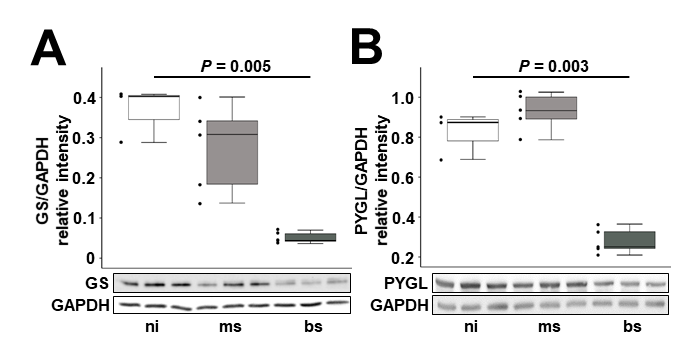


**Suppl. Figure 17: *S. mansoni* bisex infection reduced hepatic glycogen turnover.** Western blot analysis demonstrated lower hepatic expression of glycogen synthase **(A)** and hepatic glycogen phosphorylase **(B)** in bisex-*S. mansoni* infected hamsters. Representative blots are shown. Kruskall-Wallis test.

**Suppl. Figure 18**


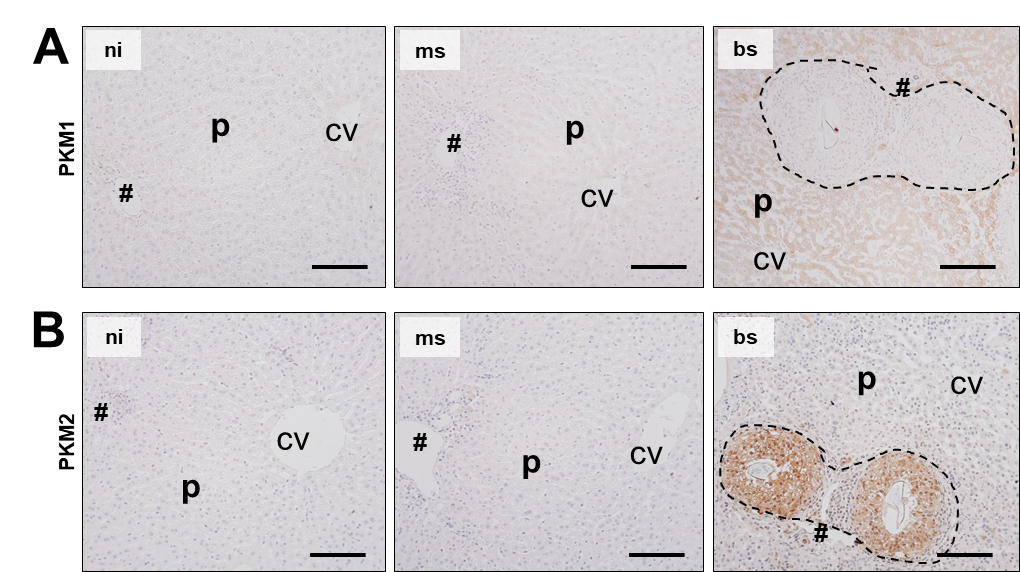


**Suppl. Figure 18: Enhanced hepatic expression pattern of PKM1 and PKM2 in *S. mansoni* infected hamsters.** While PKM1 expression was enhanced in hepatocytes (**A**), PKM2 expression was induced in granulomas (**B**) in bisex infected hamsters. p parenchyma, # portal tract, cv central vein, dashed line granuloma. Bars 100 µm.

Please note, that PKM1 and PKM2 are splicing variants of the same gene. Therefore, the results in Fig. S17A/B may suggest an alternative splicing of the PKM gene expression products in immune cells versus hepatocytes.

**Suppl. Figure 19**


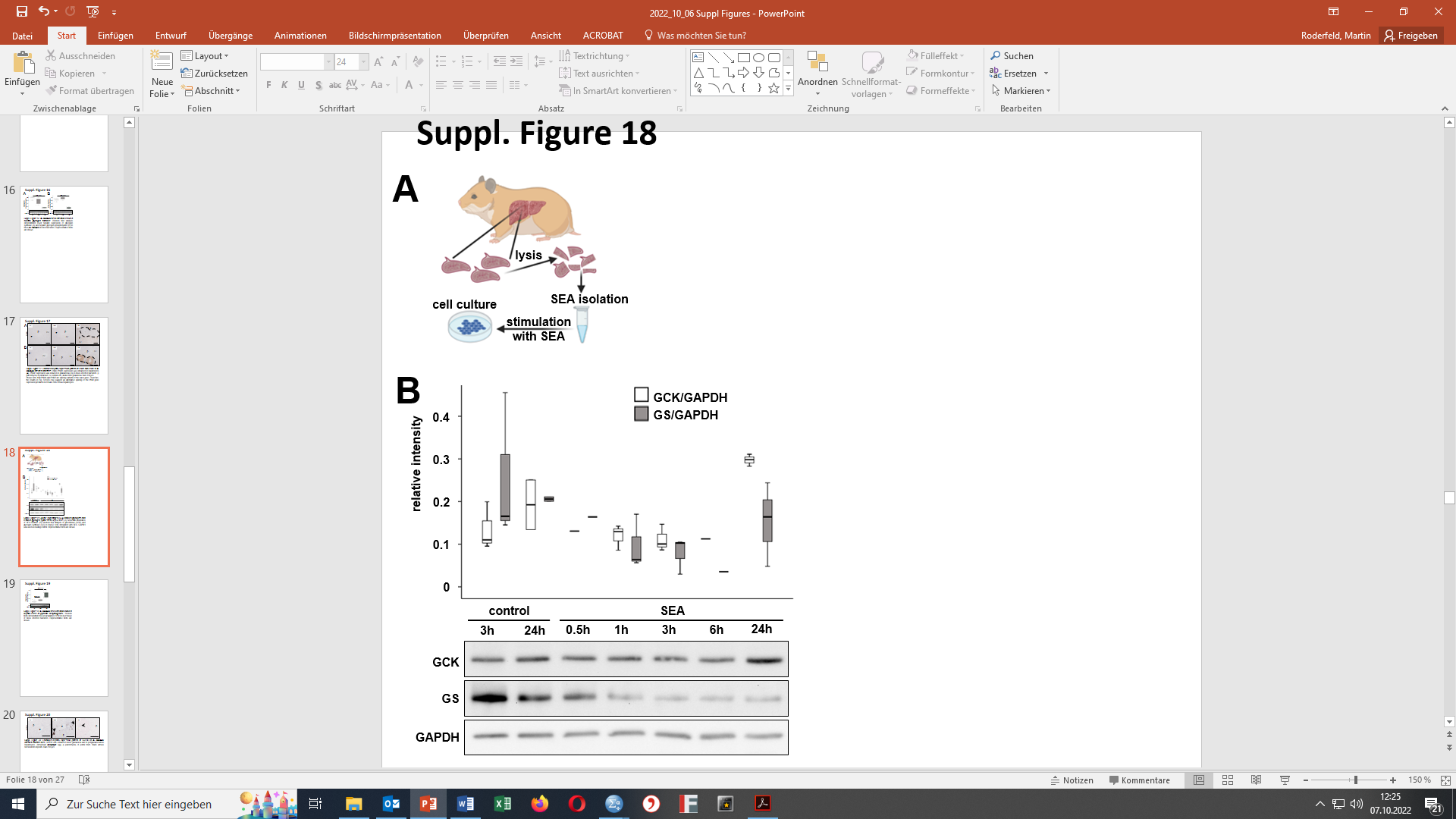


**Suppl. Figure 19: Soluble egg antigen (SEA) induced glycolysis and reduced glycogen synthesis in HepG2 cells. (A)** Schematic illustration of SEA isolation. **(B)** Western blot analysis of glucokinase (GCK) and glycogen synthase (GS) in HepG2 cells stimulated with SEA. GAPDH was used as loading control. Representative blots are shown. Kruskall-Wallis test.

**Suppl. Figure 20**


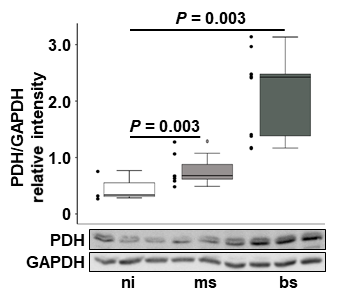


**Suppl. Figure 20: *S. mansoni* bisex infection induced hepatic levels of pyruvate dehydrogenase.** Western blots demonstrate the accumulation of PDH in liver tissue of bisex infected hamsters. Representative blots are shown. Kruskall-Wallis test.

**Suppl. Figure 21**


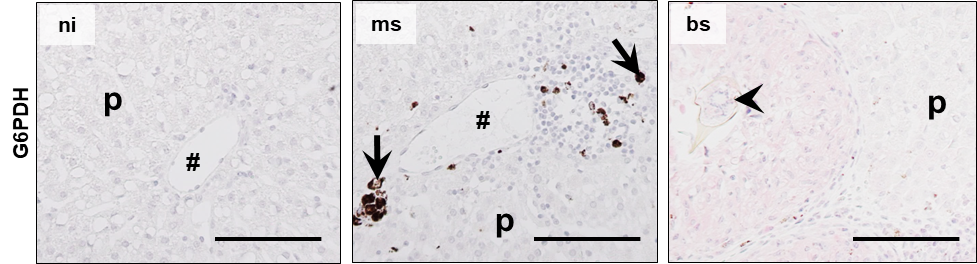


**Suppl. Figure 21: Enhanced hepatic expression pattern of G6PDH in *S. mansoni* infected hamster liver.** G6PDH was enhanced inside granuloma and in perigranulomatous hepatocytes. Arrowhead *S.mansoni* egg, p parenchyma, # portal tract, black arrows hemosiderin deposits. Bars 100 µm.

**Suppl. Figure 22**


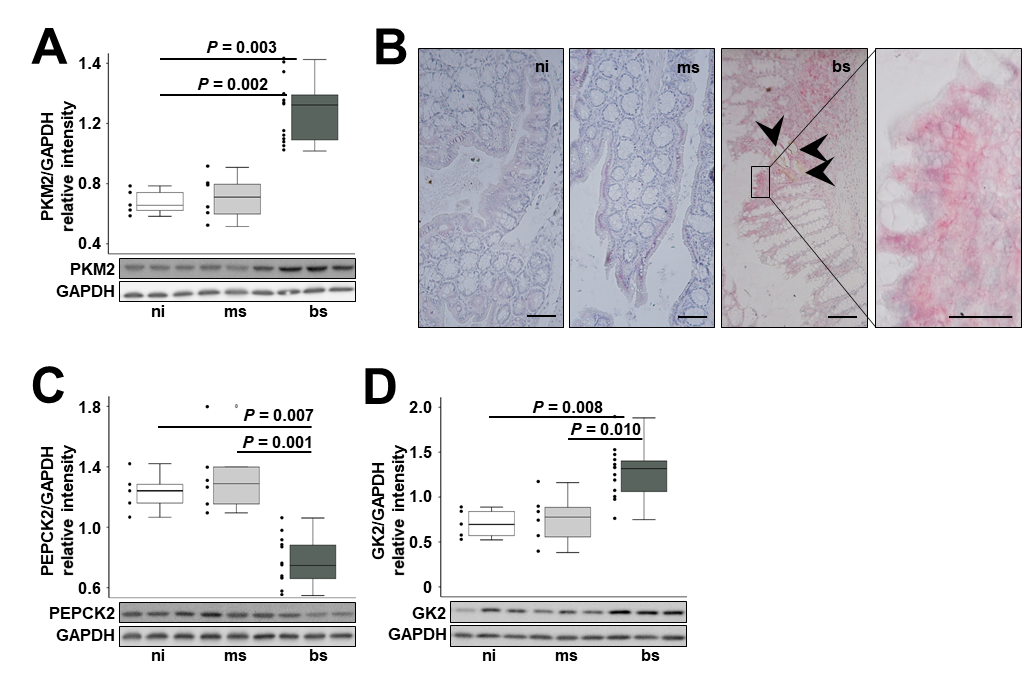


**Suppl. Figure 22: *S. mansoni* bisex infection modulated the expression of colonic PKM2, PCK2, and GK2.** Western blot (A) and immunostaining (B) demonstrated the induction of PKM2 in mucosal and submucosal cells of the colon near *S. mansoni* eggs (arrowheads, bars 200 µm and 50 µm in the magnified panel on the right). While protein expression of PEPCK2 (C) was reduced in the colon of bisex infected hamsters, GK2 was induced (D). All experiments were reproduced at least 3 times. Kruskall-Wallis test.

**Suppl. Figure 23**


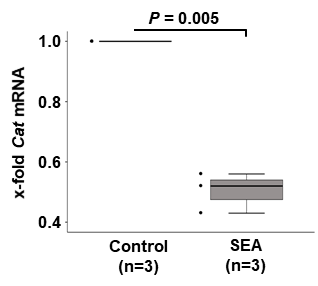


**Suppl. Figure 23: SEA stimulation decreased *catalase* mRNA level in HepG2 cells.** The presented data represent the mean of three independent experiments. Kruskall-Wallis test.

**Suppl. Figure 24**


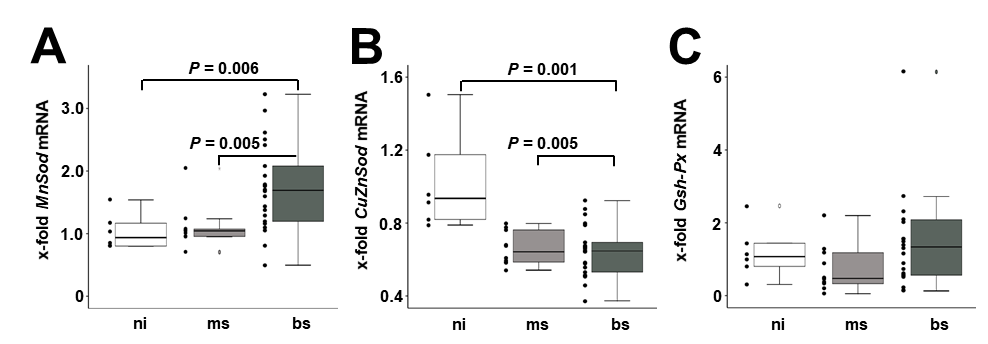


**Suppl. Figure 24: *S. mansoni* bisex infection modulated hepatic mRNA of oxidative stress markers.** qRT-PCR demonstrated the accumulation of *MnSod*-mRNA **(A)** and the decrease of *CuZnSod*-mRNA **(B)** in liver tissue of bisex infected hamsters. **(C)** *Gsh-Px* was not regulated. Representative mean data of at least three independent experiments is depicted. Kruskall-Wallis test.

**Suppl. Figure 25**


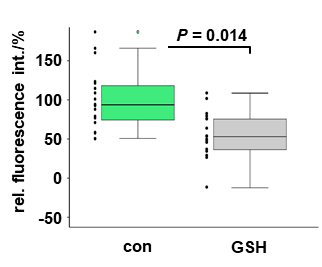


**Suppl. Figure 25: GSH decreased flOA uptake of the eggs from flOA-fed HepG2 cells in coculture.** CLSM-based quantification of fluorescence intensity of individual eggs from coculture performed with GSH (10 mM, grey bar) in comparison to the conventional experiment as a control (con, green bar). At least 10 eggs per condition were analyzed in each of two independent experiments. Kruskall-Wallis test.

**Suppl. Figure 26**


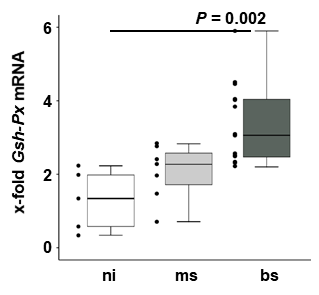


**Suppl. Figure 26: *S. mansoni* bisex infection modulated the expression of colonic *Gsh-Px*.** qRT-PCR demonstrated the induction of *Gsh-Px* in colon tissue of bisex-infected hamsters. All experiments were reproduced at least 3 times. Kruskall-Wallis test.

**Suppl. Figure 27**


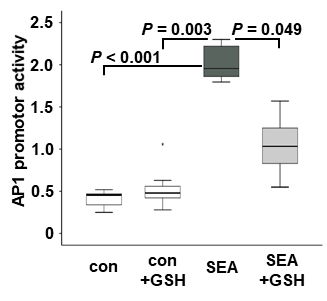


**Suppl. Figure 27: SEA activated promotor activity was reduced by GSH.** The reporter gene assay demonstrated, that SEA induced functional activation of the AP-1 promotor was diminished by the addition of GSH. All experiments were reproduced at least 3 times. Kruskall-Wallis test.

**Suppl. Figure 28**


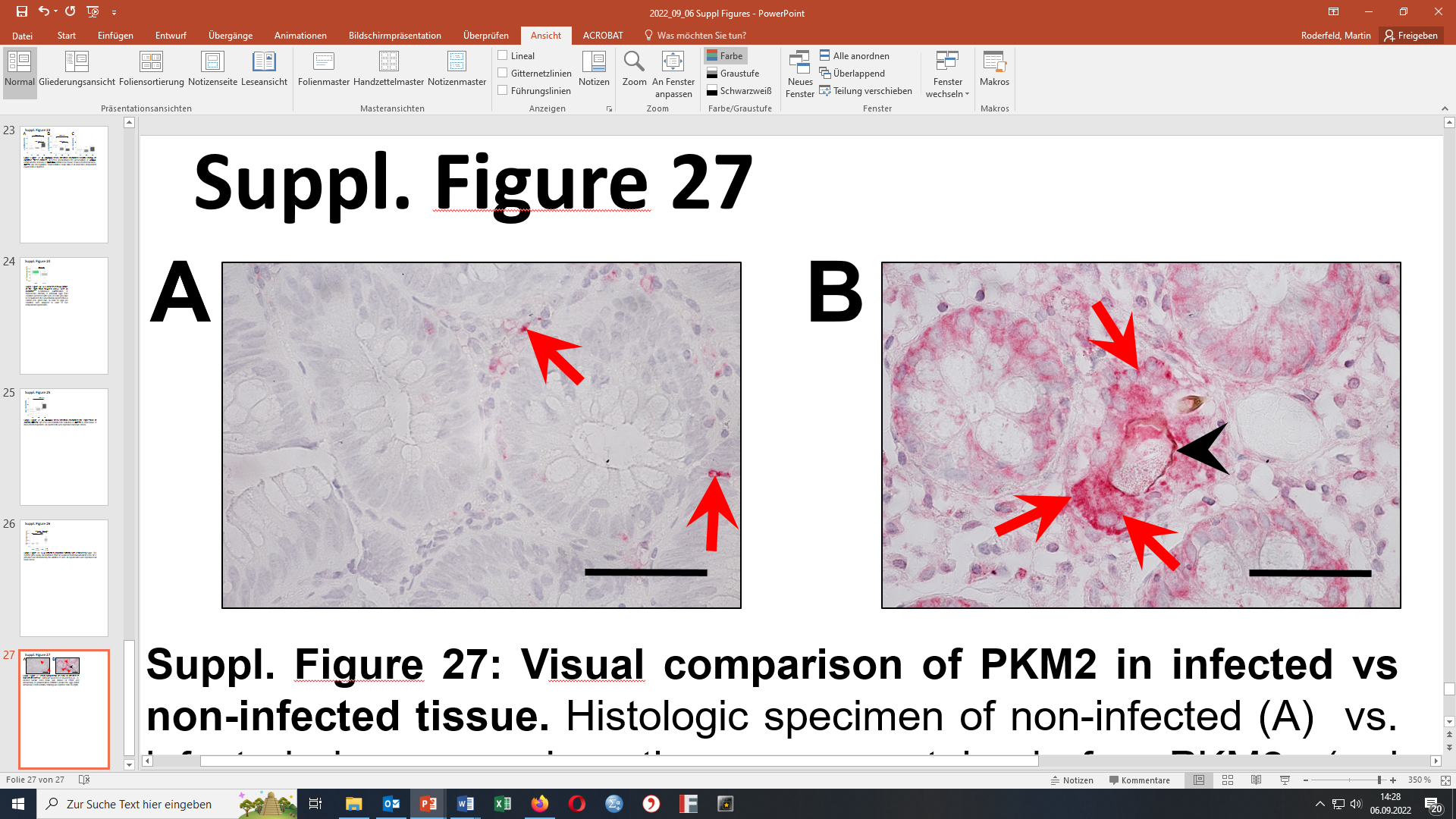


**Suppl. Figure 28:** Visual comparison of PKM2 in infected vs non-infected tissue. Histologic specimen of non-infected (A) vs. infected human colon tissue was stained for PKM2 (red arrowheads) in granulomatous infiltrates around the eggs (black arrowhead). Representative stainings are depicted, bars 50 µm.

**Supplementary CTAT Tables**

- 1. **Antibodies**

| **Name** | **Citation** | **Supplier** | **Cat no.** | **Clone no.** |
| --- | --- | --- | --- | --- |
| ADRP/Perilipin 2 | PMID: 31570772 | Proteintech | 15294-1-AP | Polycl. rabbit antibody |
| PKM2 | PMID: 15908230 | CST | 4053 | D78A4 |
| FAS | PMID: 34855620 | CST | 3189 | Polycl. rabbit antibody |
| glycogen synthase | PMID: 35563842 | CST | 3886 | 15B1 |
| PYGL | PMID: 20957198 | Novus | NBP1-86182 | Polycl. rabbit antibody |
| PKM1 | PMID: 35610475 | CST | 7067 | D30G6 |
| PKM2 | PMID: 35584006 | CST | 4053 | D78A4 |
| GCK | PMID: 34476698 | Novus | NBP1-33144 | Polycl. rabbit antibody |
| GAPDH | PMID: 30555521 | Proteintech | 60004-1-Ig | 1E6D9 |
| PDH | PMID: 26823462 | CST | 3205 | C54G1 |
| G6PDH | PMID: 35213227 | CST | 8866 | Polycl. rabbit antibody |
| PEPCK | PMID: 34954190 | B. Christ, Leipzig | homemade | Polycl. rabbit antibody |
| Phospho-c-Jun | PMID: 35896539 | CST | 3270 | D47G9 |
| c-Jun | PMID: 35896539 | CST | 9165 | 60A8 |
| Phospho-STAT3 | PMID: 35906210 | CST | 9145 | D3A7 |
| STAT3 | PMID: 35906694 | CST | 4904 | 79D7 |
| p44/42 ERK | PMID: 35306530 | CST | 4695 | 137F5 |
| Phospho-p44/42 ERK | PMID: 35306530 | CST | 4370 | D13.14.4E |
| Phospho-H2A.X | PMID: 35578032 | CST | 9718 | 20E3 |

- 1. **Cell lines**

| **Name** | **Citation** | **Supplier** | **Cat no.** | **Passage no.** | **Authentication test method** |
| --- | --- | --- | --- | --- | --- |
| HepG2 | PMID233137 | CLS | #330198 | 10-15 | Morphology |

- 1. **Organisms**

| **Name** | **Citation** | **Supplier** | **Strain** | **Sex** | **Age** | **Overall n number** |
| --- | --- | --- | --- | --- | --- | --- |
| Syrian hamsters, final hosts | PMID: 18119161 | own breeding | *Mesocricetus auratus* | female | 18 weeks | 36 |
| Schistosomes | PMID: **7477111** | Bayer AG (Monheim, Germany) | *S. mansoni* | female & male | Worms: 46-67 days | ~5.000 |
| Snail, intermediate hosts | PMID: 27499125 | own breeding | *Biomphalaria glabrata* | hermaphrodite | 1-6 months | ~20 |

- 1. **Sequence based reagents**

| **Name** | **Sequence** | **Supplier** |
| --- | --- | --- |
| *Fas* | F: 5'-CAC AGA TGA TGA CAG GAG ATG G-3'  R: 5'-TCG GAG TGA GGC TGG GTT GAT-3' | Microsynth Seqlab GmbH, Göttingen, Germany |
| *Acc1* | F: 5'-ACA CTG GCT GGC TGG ACA G-3'  R: 5'-CAC ACA ACT CCC AAC ATG GTG-3' | Microsynth Seqlab GmbH, Göttingen, Germany |
| *Cat* | F: 5'-TCC GTC CTT CAT CCA TAG C-3'  R: 5'-TGT GAG CCG TAG CCA TTC-3' | Microsynth Seqlab GmbH, Göttingen, Germany |
| *MnSod* | F: 5'-CTG GAG CCG CAC ATC AAC-3'  R: 5'-AAG AGC AAC CTG AGT CGT AAC-3' | Microsynth Seqlab GmbH, Göttingen, Germany |
| *CuZnSod* | F: 5'-TCA GGA GAG CAT TCC ATC ATT G-3'  R: 5'-TCC CAG CAT TTC CAG TCT TTG-3' | Microsynth Seqlab GmbH, Göttingen, Germany |
| *GshPx* | F: 5'-TTA CAT CGC CAA GTC GTT CTA TG-3'  R: 5'-CTG GTT GAA GTC TCT GGT AGT TG-3' | Microsynth Seqlab GmbH, Göttingen, Germany |

- 1. **Biological samples**

| **Description** | **Source** | **Identifier** |
| --- | --- | --- |
| Pseudonymized human colon samples | Dr. Senckenberg Institute of Pathology, University Hospital Frankfurt | ethics committee (AZ 05/19), ID19 H1XXX1-14 |

- 1. **Deposited data**

| **Name of repository** | **Identifier** | **Link** |
| --- | --- | --- |
|  |  |  |

- 1. **Software**

| **Software name** | **Manufacturer** | **Version** |
| --- | --- | --- |
| Lipid Match Flow | Innovative Omics | 3.5 |
| Perseus | MPI | 2.0.6.0 |
| Mirion | Institute of Inorganic and Analytical Chemistry, Justus Liebig University Giessen, Prof. Dr. Bernhard Spengler | 3.3.64.22 |
| IMARIS imaging software | Bitplane | 4.8.2 |
| Image J | NIH | 1.53a |
| SPSS | IBM | 26.0 |
| Excel Professional Plus | Microsoft, Redmond, WA, USA | 2010, 2016 |
| visionCATS | CAMAG (Muttenz, Switzerland) | 2.5 (Ultimate software package) |
| Xcalibur | Thermo Fisher Scientific (Dreieich, Germany) | 3.0.152 |
| Foundation | Thermo Fisher Scientific (Dreieich, Germany) | 3.0.152 |

- 1. **Other (e.g. drugs, proteins, vectors etc.)**

| TopFlour Oleic Acid | Avanti | SKU 810259C |
| --- | --- | --- |
| Oleic Acid | Sigma | O-1008 |
| 1,5-diaminonapthalene (DAN) | Acros Organics, Geel, Belgium | 97% |
| 2,5-dihydroxy benzoic acid (DHB) | Merck, Darmstadt, Germany | for synthesis |
| 2-propanol | Chemsolute, Renningen, Germany | for HPLC |
| acetone | VWR International, Fontenay-sous-Bois, France | HiPerSolv |
| ammonium formate | Sigma-Aldrich, Steinheim, Germany | 99.995% |
| acetonitrile | VWR International, Fontenay-sous-Bois, France | HiPerSolv |
| Eosin Y solution | Sigma-Aldrich, Steinheim, Germany |  |
| ethanol | Merck, Darmstadt, Germany | Uvasol |
| Eukitt quick hardening medium | Sigma-Aldrich, Steinheim, Germany |  |
| formic acid | Honeywell, Morris Plains, NJ, USA | for mass spectrometry |
| Mayer’s hematoxylin solution | Sigma-Aldrich, Steinheim, Germany |  |
| methanol | Merck, Darmstadt, Germany | LiChroSolv |
| Methyl-tert-butylether (MTBE) | Sigma-Aldrich, Steinheim, Germany | for HPLC |
| phosphate buffered saline (PBS) | Gibco, Carlsbad, CA, USA |  |
| trifluoro acetic acid | Merck, Darmstadt, Germany | Uvasol |
| water | VWR International, Fontenay-sous-Bois, France | HiPerSolv |
| xylene | Merck, Darmstadt, Germany | for analysis |
| Cholesterol solution 10 mg/mL | Sigma Aldrich (Darmstadt, Germany) | 471274 |
| Glyceryl trioleate | Sigma Aldrich (Darmstadt, Germany) | 41679 |
| Cholesteryl oleate | Sigma Aldrich (Darmstadt, Germany) | C9253 |
| 3-*sn*-phosphatidic acid sodium salt | Sigma Aldrich (Darmstadt, Germany) | P9511 |
| Sphingomyelin | Sigma Aldrich (Darmstadt, Germany) | 85615 |
| L-*α*-phosphatidylcholine | Sigma Aldrich (Darmstadt, Germany) | P3556 |
| L-*α*-phosphatidylethanolamine | Sigma Aldrich (Darmstadt, Germany) | P7943 |
| Primuline | Sigma Aldrich (Darmstadt, Germany) | 50% |
| Ninhydrin | Sigma Aldrich (Darmstadt, Germany) | Reagent grade |
| Aniline | Sigma Aldrich (Darmstadt, Germany) | ≥ 99.5% |
| Diphenylamine | Sigma Aldrich (Darmstadt, Germany) | ≥ 99% |
| Phosphomolybdic acid hydrate | Sigma Aldrich (Darmstadt, Germany) | Reagent grade |
| *n*-Hexane | Honeywell Riedel-de Haën (Seelze, Germany) | ≥ 97% |
| Ammonia solution | Honeywell Riedel-de Haën (Seelze, Germany) | 25%, p.a. |
| *o*-Phosphoric acid | Th. Geyer (Renningen, Germany) | 85% |
| 2-Propanol | Th. Geyer (Renningen, Germany) | ≥ 99.8% |
| Chloroform | AppliChem GmbH (Darmstadt, Germany) | > 99.0% |
| Methanol | VWR (Darmstadt, Germany) | ≥ 99.8% |
| Ethanol | Fisher Scientific (Schwerte, Germany) | ≥ 99.8% |
| Diethyl ether | Acros Organics (New Jersey, NJ, USA) | > 99% |
| Glacial acetic acid | Carl Roth (Karlsruhe, Germany) | 100%, p.a. |

- 1. **Please provide the details of the corresponding methods author for the manuscript:**

| PD Dr. Martin Roderfeld  Justus-Liebig University Giessen  martin.roderfeld@innere.med.uni-giessen.de |
| --- |

**2.0 Please confirm for randomised controlled trials all versions of the clinical protocol are included in the submission. These will be published online as supplementary information.**

|  |
| --- |

|  | | **Oleic acid** | | **Triolein** | | **Cholesterol** | | **Cholesteryl oleate** | | **Phosphatidyl-ethanolamine** | | **Phosphatidic acid** | | **Sphingomyelin** | | **Phosphatidyl-choline** | |
| --- | --- | --- | --- | --- | --- | --- | --- | --- | --- | --- | --- | --- | --- | --- | --- | --- | --- |
| **Group** | **Sample track** | **Mean content [mg/g]** | **Reprodu-cibility**  **[%RSD]** | **Mean content [mg/g]** | **Reprodu-cibility**  **[%RSD]** | **Mean content [mg/g]** | **Reprodu-cibility**  **[%RSD]** | **Mean content [mg/g]** | **Reprodu-cibility**  **[%RSD]** | **Mean content [mg/g]** | **Reprodu-cibility**  **[%RSD]** | **Mean content [mg/g]** | **Reprodu-cibility**  **[%RSD]** | **Mean content [mg/g]** | **Reprodu-cibility**  **[%RSD]** | **Mean content [mg/g]** | **Reprodu-cibility**  **[%RSD]** |
| ni | 1 | 1.4 | 12.7 | 1.7 | 9.7 | 0.9 | 0.5 | <LOD |  | 18.5 | 3.6 | <LOQ |  | 0.6 | 0.1 | 9.6 | 1.1 |
| ni | 2 | 1.3 | 6.7 | 2.3 | 4.0 | 0.6 | 17.6 | <LOD |  | 18.2 | 1.5 | 0.3 | 1.2 | 0.6 | 10.9 | 10.6 | 1.8 |
| ni | 3 | 1.2 | 17.5 | 1.2 | 6.0 | 0.7 | 15.6 | - |  | 21.0 | 3.6 | 0.2 | 6.7 | 0.8 | 10.5 | 11.1 | 2.2 |
| ms | 4 | 1.3 | 12.7 | 1.1 | 19.3 | 1.0 | 0.5 | 21.2 | 4.9 | 19.1 | 6.4 | <LOQ |  | 0.7 | 12.1 | 9.3 | 2.6 |
| ms | 5 | 1.6 | 12.7 | 1.0 | 28.8 | 0.9 | 0.0 | 16.4 | 3.1 | 19.9 | 0.1 | <LOQ |  | 0.9 | 6.7 | 10.0 | 0.3 |
| ms | 6 | 1.8 | 5.7 | 1.7 | 13.9 | 0.9 | 4.3 | 2.4 | 4.3 | 19.2 | 2.0 | <LOQ |  | 0.7 | 5.5 | 9.5 | 5.2 |
| ms | 7 | 1.6 | 4.4 | 1.5 | 12.3 | 1.1 | 7.1 | <LOD |  | 21.8 | 5.7 | <LOQ |  | 0.8 | 0.2 | 10.5 | 3.7 |
| ms | 8 | 1.5 | 5.5 | 1.0 | 19.8 | 1.0 | 9.6 | <LOD |  | 20.2 | 6.4 | <LOQ |  | 0.8 | 6.4 | 9.7 | 0.2 |
| ms | 9 | 1.4 | 5.7 | 1.1 | 15.2 | 0.7 | 9.7 | <LOD |  | 18.1 | 5.4 | 0.2 | 2.8 | 0.8 | 3.3 | 9.4 | 0.3 |
| bs | 10 | 1.6 | 9.6 | 1.9 | 4.3 | 0.9 | 1.9 | <LOD |  | 22.4 | 0.1 | 0.3 | 7.0 | 1.1 | 2.2 | 10.9 | 7.6 |
| bs | 11 | 1.8 | 9.5 | 1.5 | 14.4 | 1.0 | 12.1 | <LOD |  | 21.0 | 6.5 | 0.3 | 1.6 | 1.1 | 4.6 | 9.9 | 10.4 |
| bs | 12 | 1.8 | 10.5 | 1.2 | 20.0 | 1.0 | 12.6 | <LOD |  | 18.4 | 7.8 | 0.2 | 19.6 | 0.9 | 6.0 | 9.2 | 8.5 |
| bs | 13 | 1.9 | 7.9 | 1.5 | 18.7 | 1.1 | 14.7 | <LOD |  | 19.4 | 8.4 | 0.2 | 21.9 | 0.8 | 15.1 | 9.3 | 6.8 |
| bs | 14 | 1.9 | 7.1 | 1.1 | 21.1 | 1.1 | 17.2 | <LOD |  | 19.5 | 7.1 | <LOQ |  | 1.0 | 0.5 | 10.1 | 6.6 |
| bs | 15 | 1.9 | 5.1 | 3.8 | 3.4 | 1.3 | 18.5 | <LOD |  | 17.5 | 14.4 | <LOQ |  | 0.8 | 0.4 | 9.2 | 7.2 |

**Suppl. Table 1: Quantification results and reproducibilities (*n* = 3) determined via HPTLC-FLD.**
