# Supplementary material for: Metabolic reprogramming of hepatocytes by Schistosoma mansoni eggs
Source: JHEP Rep. 2022 Nov 7;5(2):100625. doi: 10.1016/j.jhepr.2022.100625 (PMC9800334; doi:10.1016/j.jhepr.2022.100625)
Supplement: Multimedia component 2 [file mmc2.docx]

**Journal of Hepatology**

**CTAT methods**

Tables for a “Complete, Transparent, Accurate and Timely account” (CTAT) are now mandatory for all revised submissions. The aim is to enhance the reproducibility of methods.

- Only include the parts relevant to your study
- Refer to the CTAT in the main text as ‘Supplementary CTAT Table’
- Do not add subheadings
- Add as many rows as needed to include all information
- Only include one item per row

**If the CTAT form is not relevant to your study, please outline the reasons why:**

|  |
| --- |

- 1. **Antibodies**

| **Name** | **Citation** | **Supplier** | **Cat no.** | **Clone no.** |
| --- | --- | --- | --- | --- |
| ADRP/Perilipin 2 | PMID: 31570772 | Proteintech | 15294-1-AP | Polycl. rabbit antibody |
| PKM2 | PMID: 15908230 | CST | 4053 | D78A4 |
| FAS | PMID: 34855620 | CST | 3189 | Polycl. rabbit antibody |
| glycogen synthase | PMID: 35563842 | CST | 3886 | 15B1 |
| PYGL | PMID: 20957198 | Novus | NBP1-86182 | Polycl. rabbit antibody |
| PKM1 | PMID: 35610475 | CST | 7067 | D30G6 |
| PKM2 | PMID: 35584006 | CST | 4053 | D78A4 |
| GCK | PMID: 34476698 | Novus | NBP1-33144 | Polycl. rabbit antibody |
| GAPDH | PMID: 30555521 | Proteintech | 60004-1-Ig | 1E6D9 |
| PDH | PMID: 26823462 | CST | 3205 | C54G1 |
| G6PDH | PMID: 35213227 | CST | 8866 | Polycl. rabbit antibody |
| PEPCK | PMID: 34954190 | B. Christ, Leipzig | homemade | Polycl. rabbit antibody |
| Phospho-c-Jun | PMID: 35896539 | CST | 3270 | D47G9 |
| c-Jun | PMID: 35896539 | CST | 9165 | 60A8 |
| Phospho-STAT3 | PMID: 35906210 | CST | 9145 | D3A7 |
| STAT3 | PMID: 35906694 | CST | 4904 | 79D7 |
| p44/42 ERK | PMID: 35306530 | CST | 4695 | 137F5 |
| Phospho-p44/42 ERK | PMID: 35306530 | CST | 4370 | D13.14.4E |
| Phospho-H2A.X | PMID: 35578032 | CST | 9718 | 20E3 |

- 1. **Cell lines**

| **Name** | **Citation** | **Supplier** | **Cat no.** | **Passage no.** | **Authentication test method** |
| --- | --- | --- | --- | --- | --- |
| HepG2 | PMID233137 | CLS | #330198 | 10-15 | Morphology |

- 1. **Organisms**

| **Name** | **Citation** | **Supplier** | **Strain** | **Sex** | **Age** | **Overall n number** |
| --- | --- | --- | --- | --- | --- | --- |
| Syrian hamsters, final hosts | PMID: 18119161 | own breeding | *Mesocricetus auratus* | female | 18 weeks | 36 |
| Schistosomes | PMID: **7477111** | Bayer AG (Monheim, Germany) | *S. mansoni* | female & male | Worms: 46-67 days | ~5.000 |
| Snail, intermediate hosts | PMID: 27499125 | own breeding | *Biomphalaria glabrata* | hermaphrodite | 1-6 months | ~20 |

- 1. **Sequence based reagents**

| **Name** | **Sequence** | **Supplier** |
| --- | --- | --- |
| *Fas* | F: 5'-CAC AGA TGA TGA CAG GAG ATG G-3'  R: 5'-TCG GAG TGA GGC TGG GTT GAT-3' | Microsynth Seqlab GmbH, Göttingen, Germany |
| *Acc1* | F: 5'-ACA CTG GCT GGC TGG ACA G-3'  R: 5'-CAC ACA ACT CCC AAC ATG GTG-3' | Microsynth Seqlab GmbH, Göttingen, Germany |
| *Cat* | F: 5'-TCC GTC CTT CAT CCA TAG C-3'  R: 5'-TGT GAG CCG TAG CCA TTC-3' | Microsynth Seqlab GmbH, Göttingen, Germany |
| *MnSod* | F: 5'-CTG GAG CCG CAC ATC AAC-3'  R: 5'-AAG AGC AAC CTG AGT CGT AAC-3' | Microsynth Seqlab GmbH, Göttingen, Germany |
| *CuZnSod* | F: 5'-TCA GGA GAG CAT TCC ATC ATT G-3'  R: 5'-TCC CAG CAT TTC CAG TCT TTG-3' | Microsynth Seqlab GmbH, Göttingen, Germany |
| *GshPx* | F: 5'-TTA CAT CGC CAA GTC GTT CTA TG-3'  R: 5'-CTG GTT GAA GTC TCT GGT AGT TG-3' | Microsynth Seqlab GmbH, Göttingen, Germany |

- 1. **Biological samples**

| **Description** | **Source** | **Identifier** |
| --- | --- | --- |
| Pseudonymized human colon samples | Dr. Senckenberg Institute of Pathology, University Hospital Frankfurt | ethics committee (AZ 05/19), ID19 H1XXX1-14 |

- 1. **Deposited data**

| **Name of repository** | **Identifier** | **Link** |
| --- | --- | --- |
|  |  |  |

- 1. **Software**

| **Software name** | **Manufacturer** | **Version** |
| --- | --- | --- |
| Lipid Match Flow | Innovative Omics | 3.5 |
| Perseus | MPI | 2.0.6.0 |
| Mirion | Institute of Inorganic and Analytical Chemistry, Justus Liebig University Giessen, Prof. Dr. Bernhard Spengler | 3.3.64.22 |
| IMARIS imaging software | Bitplane | 4.8.2 |
| Image J | NIH | 1.53a |
| SPSS | IBM | 26.0 |
| Excel Professional Plus | Microsoft, Redmond, WA, USA | 2010, 2016 |
| visionCATS | CAMAG (Muttenz, Switzerland) | 2.5 (Ultimate software package) |
| Xcalibur | Thermo Fisher Scientific (Dreieich, Germany) | 3.0.152 |
| Foundation | Thermo Fisher Scientific (Dreieich, Germany) | 3.0.152 |

- 1. **Other (e.g. drugs, proteins, vectors etc.)**

| TopFlour Oleic Acid | Avanti | SKU 810259C |
| --- | --- | --- |
| Oleic Acid | Sigma | O-1008 |
| 1,5-diaminonapthalene (DAN) | Acros Organics, Geel, Belgium | 97% |
| 2,5-dihydroxy benzoic acid (DHB) | Merck, Darmstadt, Germany | for synthesis |
| 2-propanol | Chemsolute, Renningen, Germany | for HPLC |
| acetone | VWR International, Fontenay-sous-Bois, France | HiPerSolv |
| ammonium formate | Sigma-Aldrich, Steinheim, Germany | 99.995% |
| acetonitrile | VWR International, Fontenay-sous-Bois, France | HiPerSolv |
| Eosin Y solution | Sigma-Aldrich, Steinheim, Germany |  |
| ethanol | Merck, Darmstadt, Germany | Uvasol |
| Eukitt quick hardening medium | Sigma-Aldrich, Steinheim, Germany |  |
| formic acid | Honeywell, Morris Plains, NJ, USA | for mass spectrometry |
| Mayer’s hematoxylin solution | Sigma-Aldrich, Steinheim, Germany |  |
| methanol | Merck, Darmstadt, Germany | LiChroSolv |
| Methyl-tert-butylether (MTBE) | Sigma-Aldrich, Steinheim, Germany | for HPLC |
| phosphate buffered saline (PBS) | Gibco, Carlsbad, CA, USA |  |
| trifluoro acetic acid | Merck, Darmstadt, Germany | Uvasol |
| water | VWR International, Fontenay-sous-Bois, France | HiPerSolv |
| xylene | Merck, Darmstadt, Germany | for analysis |
| Cholesterol solution 10 mg/mL | Sigma Aldrich (Darmstadt, Germany) | 471274 |
| Glyceryl trioleate | Sigma Aldrich (Darmstadt, Germany) | 41679 |
| Cholesteryl oleate | Sigma Aldrich (Darmstadt, Germany) | C9253 |
| 3-*sn*-phosphatidic acid sodium salt | Sigma Aldrich (Darmstadt, Germany) | P9511 |
| Sphingomyelin | Sigma Aldrich (Darmstadt, Germany) | 85615 |
| L-*α*-phosphatidylcholine | Sigma Aldrich (Darmstadt, Germany) | P3556 |
| L-*α*-phosphatidylethanolamine | Sigma Aldrich (Darmstadt, Germany) | P7943 |
| Primuline | Sigma Aldrich (Darmstadt, Germany) | 50% |
| Ninhydrin | Sigma Aldrich (Darmstadt, Germany) | Reagent grade |
| Aniline | Sigma Aldrich (Darmstadt, Germany) | ≥ 99.5% |
| Diphenylamine | Sigma Aldrich (Darmstadt, Germany) | ≥ 99% |
| Phosphomolybdic acid hydrate | Sigma Aldrich (Darmstadt, Germany) | Reagent grade |
| *n*-Hexane | Honeywell Riedel-de Haën (Seelze, Germany) | ≥ 97% |
| Ammonia solution | Honeywell Riedel-de Haën (Seelze, Germany) | 25%, p.a. |
| *o*-Phosphoric acid | Th. Geyer (Renningen, Germany) | 85% |
| 2-Propanol | Th. Geyer (Renningen, Germany) | ≥ 99.8% |
| Chloroform | AppliChem GmbH (Darmstadt, Germany) | > 99.0% |
| Methanol | VWR (Darmstadt, Germany) | ≥ 99.8% |
| Ethanol | Fisher Scientific (Schwerte, Germany) | ≥ 99.8% |
| Diethyl ether | Acros Organics (New Jersey, NJ, USA) | > 99% |
| Glacial acetic acid | Carl Roth (Karlsruhe, Germany) | 100%, p.a. |

- 1. **Please provide the details of the corresponding methods author for the manuscript:**

| PD Dr. Martin Roderfeld  Justus-Liebig University Giessen  martin.roderfeld@innere.med.uni-giessen.de |
| --- |

**2.0 Please confirm for randomised controlled trials all versions of the clinical protocol are included in the submission. These will be published online as supplementary information.**

|  |
| --- |
